# Dietary sulfur amino acid restriction improves metabolic health by reducing fat mass

**DOI:** 10.1093/lifemeta/loaf009

**Published:** 2025-03-07

**Authors:** Chenhao Xin, Mingcheng Cai, Qianxi Jia, Rong Huang, Rui Li, Junyao Wang, Zi Li, Qiang Zhao, Tianyi Liu, Weidong Zhuang, Jinyu Zhou, Shengxian Li, Yongzhen Tao, Lin Wang, Lifeng Yang

**Affiliations:** Shanghai Institute of Nutrition and Health, University of Chinese Academy of Sciences, Chinese Academy of Sciences, Shanghai 200031, China; Shanghai Institute of Nutrition and Health, University of Chinese Academy of Sciences, Chinese Academy of Sciences, Shanghai 200031, China; Shanghai Institute of Nutrition and Health, University of Chinese Academy of Sciences, Chinese Academy of Sciences, Shanghai 200031, China; Department of Endocrinology and Metabolism, Renji Hospital, School of Medicine, Shanghai Jiao Tong University, Shanghai 200127, China; Shanghai Institute of Nutrition and Health, University of Chinese Academy of Sciences, Chinese Academy of Sciences, Shanghai 200031, China; Shanghai Institute of Nutrition and Health, University of Chinese Academy of Sciences, Chinese Academy of Sciences, Shanghai 200031, China; Shanghai Institute of Nutrition and Health, University of Chinese Academy of Sciences, Chinese Academy of Sciences, Shanghai 200031, China; Shanghai Institute of Nutrition and Health, University of Chinese Academy of Sciences, Chinese Academy of Sciences, Shanghai 200031, China; Shanghai Institute of Nutrition and Health, University of Chinese Academy of Sciences, Chinese Academy of Sciences, Shanghai 200031, China; Shanghai Institute of Nutrition and Health, University of Chinese Academy of Sciences, Chinese Academy of Sciences, Shanghai 200031, China; State Key Laboratory of Common Mechanism Research for Major Disease, Institute of Basic Medical Sciences, Chinese Academy of Medical Sciences and Peking Union Medical College, Beijing 100730, China; Organ Transplant Center, Shanghai Changzheng Hospital (Second Affiliated Hospital of Naval Medical University), Shanghai 200003, China; Shanghai Institute of Nutrition and Health, University of Chinese Academy of Sciences, Chinese Academy of Sciences, Shanghai 200031, China; State Key Laboratory of Common Mechanism Research for Major Disease, Institute of Basic Medical Sciences, Chinese Academy of Medical Sciences and Peking Union Medical College, Beijing 100730, China; Shanghai Institute of Nutrition and Health, University of Chinese Academy of Sciences, Chinese Academy of Sciences, Shanghai 200031, China

**Keywords:** diet intervention, sulfur amino acid, fat loss, isotope tracing, metabolic flux, glucose and lipid metabolism

## Abstract

Diet interventions such as calorie restriction or time-restricted feeding offer potential for weight management, but long-term success is often hindered by poor adherence due to the rewarding effects of sugars. In this study, we demonstrate that sulfur amino acid restriction (SAAR) diets promote rapid fat loss without impairing appetite and physiological locomotion, outperforming diets with restricted branched-chain amino acids. Weekly cycling of SAAR diets preserves metabolic benefits, such as reduced fat mass and improved glucose sensitivity. Metabolic analysis and *in vivo* isotope tracing revealed a shift toward carbohydrate oxidation in white and brown adipose tissue (WAT and BAT), and liver during the SAAR diet refeeding state, leading to decreased *de novo* lipogenesis. Enhanced lipolysis and fatty acid oxidation were observed in the heart, brain, BAT, lungs, etc. The reintroduction of methionine or cystine negated these metabolic benefits. Further ^13^C and ^2^H tracing experiments indicated that cystine, rather than its derivatives like taurine or H_2_S, directly regulates adiposity. In a high-fat diet model, SAAR diet led to sustained fat mass reduction, regardless of the timing of intervention. Additionally, cystine levels correlated positively with body mass index (BMI) and total triglycerides in diabetic patients. Our findings highlight SAAR diet as a promising strategy for long-term weight control by modulating systemic glucose and lipid metabolism homeostasis.

## Introduction

Obesity is considered a chronic, complex disease due to excessive fat accumulation that raises the risk of metabolic syndromes such as type 2 diabetes, fatty liver diseases, cardiovascular diseases, various cancers, etc. [1–4]. Over the last 30 years, obesity prevalence has surged to pandemic levels, affecting 16% of the global adult population. The number has more than doubled since 1990, and that has quadrupled among adolescents. Common therapeutic strategies like exercise, sodium-dependent glucose transporters 2 (SGLT-2) inhibitors, bariatric surgery, and glucagon-like peptide (GLP)-1 receptor agonists could reduce circulatory glucose levels and promote short-term weight loss [5–8]. However, effective weight management is a life-long process. Failure to maintain a healthy lifestyle after treatment often leads to weight regain and severe health issues. For instance, after discontinuous semaglutide treatment, patients regained two-thirds of their previous weight loss and reverted to the baseline of cardiometabolic variables [9]. Thus, there is a growing need for sustainable, long-term lifestyle changes.

Nutrient interventions offer a promising avenue for tackling obesity. Calorie restriction or time-restricted feeding regimens help reduce energy intake, but adherence is challenging and weight regain is common when these practices are discontinued [10–12]. Similarly, a low glycemic index (LGI) diet can aid in weight control and diabetes management, yet their strict avoidance of sugars leads to poor compliance due to the rewarding nature of sweet tastes [13, 14]. Artificial sweeteners, often used as sugar substitutes, have been shown to induce glucose intolerance and increase the risk of type 2 diabetes [15]. A more effective strategy could involve modulating energy expenditure (EE) by adjusting the intake of specific nutrients. For example, branched-chain amino acids (BCAAs) have been implicated in obesity, with studies showing that high levels of BCAAs can induce insulin resistance when combined with a high-fat diet [16–19]. Conversely, a diet low in BCAAs has been shown to increase EE, improve glucose sensitivity, and induce weight loss. Interestingly, valine and isoleucine, rather than leucine, seem to drive these benefits [20]. However, the precise mechanisms and the organs involved in this BCAA-mediated metabolic regulation remain unclear [21].

Sulfur amino acids (SAAs) also play a key role in metabolic health. Methionine restriction has been shown to promote longevity and improve metabolic health, with short-term methionine removal [22–24]. Cyst(e)ine, however, has different metabolic effects depending on the models studied. For example, dietary cysteine can induce fat loss by suppressing feeding behavior in mice with 60 days of cysteine gavage, or dietary addition of cysteine into sucrose for fruit flies could significantly induce fat loss via suppressive action of neuropeptide FMRFamide (FMRFa)/neuropeptide FF (NPFF) on feeding behavior [25]. On the contrary, a cross-sectional epidemiological study uncovers a positive correlation of circulatory cyst(e)ine level with triglycerides (TGs) in humans [26, 27]. Mechanistic study shows that cysteine restriction could shunt non-glucose intermediates to serine biosynthesis from glycerol synthesis, hypothesized from the expression of related genes in the liver [26]. Therefore, how to manipulate the composition of diet and which method outweighs in fighting against obesity remains elusive.

Systemic lipid homeostasis relies on a balance between dietary intake, *de novo* lipogenesis, lipolysis, and fatty acid oxidation. *In vivo* isotope tracing has advanced our understanding of how different organs regulate lipid metabolism. Studies show that more than 80% of body fat comes from dietary sources, with the liver and brown adipose tissue (BAT) synthesizing new fat from glucose, lactate, and acetate as carbon sources and glucose/serine-derived NADPH as reducing equivalent sources [28, 29]. For white adipose tissue (WAT) differentiation, increased BCAA catabolic flux for lipid synthesis was observed to partially replace fluxes from glucose and glutamine to lipid [30]. During fasting, lipolysis in WAT increases circulatory levels of free fatty acids, and fluxes of fatty acid oxidation are enhanced in most organs, particularly in the skeletal muscle, heart, and BAT [31]. Thus, figuring out the metabolic homeostasis of glucose and lipids via *in vivo* isotope tracing could enhance our understanding of the mechanism of weight control from a metabolic perspective.

In this study, we compared the effects of restricting BCAAs and SAAs in the diet and found that SAA restriction led to more significant fat loss and improved glucose sensitivity. These benefits were driven by changes in glucose and lipid metabolism, including alterations in lipogenesis, lipolysis, and fatty acid oxidation. Mechanistically, cystine, rather than methionine, or their products (e.g. taurine, H_2_S, coenzyme A (CoA), and glutathione for reactive oxygen species (ROS) clearance), plays a crucial role in regulating adiposity, as supported by findings from diabetic patients.

## Results

### SAA restriction induces weight loss and enhances glucose sensitivity without impairment of locomotion and appetite

**Figure 1:**
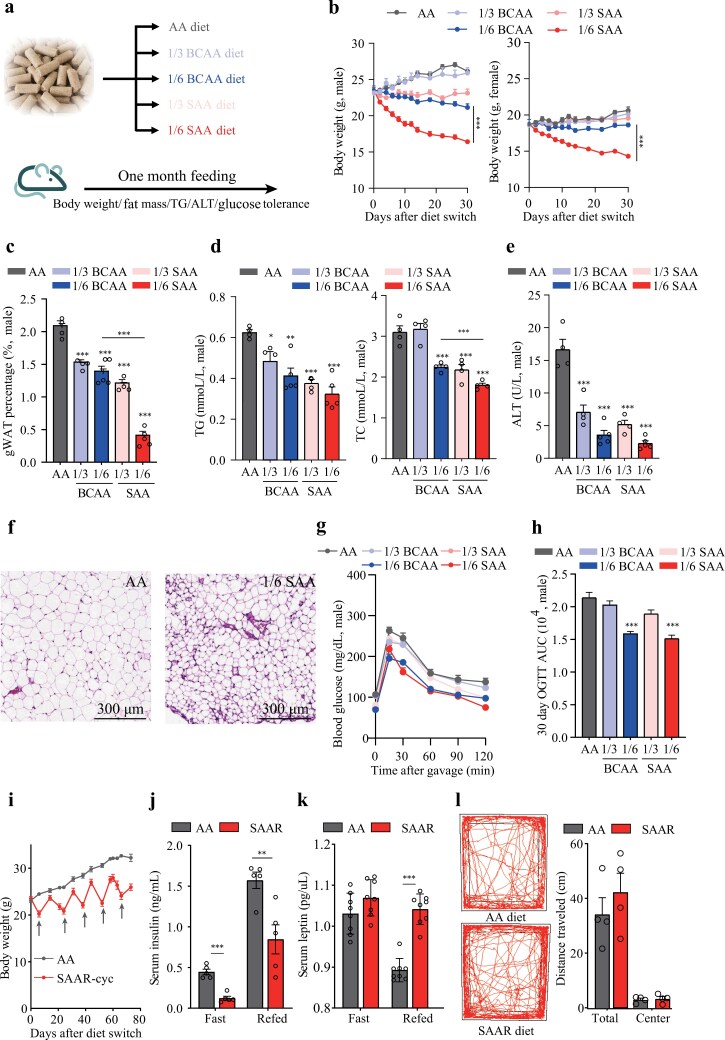
Limitation of SAA intake leads to rapid weight loss and enhances glucose sensitivity in both sexes. (a) Scheme of diet formulation with BCAA or SAA restriction. (b) Weights of mice fed with different diets. Male: AA, *n* = 4; 1/3 BCAA, *n* = 4; 1/3 SAA, *n* = 4; 1/6 BCAA, *n* = 5; 1/6 SAA, *n* = 5. Female: AA, *n* = 4; 1/3 BCAA, *n* = 4; 1/3 SAA, *n* = 4; 1/6 BCAA, *n* = 4; 1/6 SAA, *n* = 5. (c) Weight percentage of gonadal fat in mice fed with different diets. Male: AA, *n* = 4; 1/3 BCAA, *n* = 4; 1/3 SAA, *n* = 4; 1/6 BCAA, *n* = 5; 1/6 SAA, *n* = 5. (d) Circulatory levels of TG and TC in male mice fed with different diets. For TG, AA, *n* = 4; 1/3 BCAA, *n* = 4; 1/3 SAA, *n* = 4; 1/6 BCAA, *n* = 4; 1/6 SAA, *n* = 5. For TC, all groups, *n* = 4. (e) Circulatory ALT levels in male mice fed with different diets. AA, *n* = 4; 1/3 BCAA, *n* = 4; 1/3 SAA, *n* = 4; 1/6 BCAA, *n* = 5; 1/6 SAA, *n* = 5. (f) Hematoxylin and eosin (H&E) staining of gWAT from male mice fed with different diets. (g and h) Circulatory glucose levels and its corresponding area under the curve in male mice gavaged with 1 g/kg glucose after 16 h of fasting. Male: AA, *n* = 4; 1/3 BCAA, *n* = 4; 1/3 SAA, *n* = 4; 1/6 BCAA, *n* = 4; 1/6 SAA, *n* = 5. Statistical differences were calculated by one-way ANOVA with Dunnett’s correction in (h). (i) Weights of mice subjected to dietary cycling and those continuously fed with AA diet. AA, *n* = 4; SAAR-cyc, *n* = 5. (j) Circulatory insulin levels in fasting and refed periods. *n* = 5. (k) Circulatory leptin levels in fasting and refed periods. *n* = 8. (l) The traveled distances of mice fed with AA or SAAR diets within two specific zones of the open-field experiment. *n* = 4. Data are expressed as mean ± SEM and statistical differences were calculated by unpaired two-tail *t*-test if unspecified. ^*^P < 0.05; ^**^P < 0.01; ^***^P < 0.001.

To investigate the impact of BCAAs and SAAs on weight control, we formulated amino acid diets (AA diets) based on the casein composition of a normal chow diet (Supplementary Table S1). We then created the low BCAA diets by reducing BCAA levels to 1/3 and 1/6 of those in the formulated AA diet. Similarly, we developed the sulfur amino acid restriction (SAAR) diets by decreasing methionine to 1/3 and 1/6 without cystine supplementation, marked as 1/3 BCAA, 1/6 BCAA, 1/3 SAA, and 1/6 SAA, respectively (Fig. 1a). After feeding C57BL/6J mice with these diets for one month, we observed that the 1/3 BCAA diet maintained weight gain similar to mice in AA diet for both sexes (Fig. 1b). The 1/6 BCAA diet resulted in gradual weight loss in male mice but maintained weight in females (Fig. 1b). In contrast, the 1/3 SAA diet only slightly affected male body weight, while the 1/6 SAA diet caused rapid weight loss in both sexes, without impacting daily food intake and feces output, but leading to increased water intake and urine output (Supplementary Fig. S1a and b). Quantification of gonadal WAT (gWAT) weight after one month of feeding revealed that the 1/3 BCAA, 1/6 BCAA, and 1/3 SAA diets reduced gWAT percentage in males, but only the 1/6 SAA diet significantly decreased fat mass in both sexes (Fig. 1c; Supplementary Fig. S1c). Similar trends were observed in circulating levels of total TG and total cholesterol (TC), which decreased more significantly in males than females (Fig. 1d; Supplementary Fig. S1d). The observed differences may be attributed to variations in the mechanistic target of rapamycin complex 1 (mTORC1) activity, hormonal counteract effects, or differences in body composition across sexes [32, 33]. However, the consistent effects of the 1/6 SAA diet across both sexes suggest that the SAAR diet generally influences fat metabolism. To validate it, we measured the organ weights for mice with a 1/6 SAA diet and found that weight loss occurred in the gWAT, inguinal WAT (iWAT), liver, kidney, and pancreas, but not the muscles (Supplementary Fig. S1e). Furthermore, we found that alanine aminotransferase (ALT) level, a marker of liver damage, reduced in both BCAA and SAAR diets, with the lowest levels observed in the 1/6 SAA diet, indicating protective effects (Fig. 1e; Supplementary Fig. S1f). Histological staining further confirmed the smallest adipocyte size in gWAT and the lowest fat deposit in the liver for male mice fed with 1/6 SAA diet after 30 days, while Masson’s trichrome (Masson) staining showed that 1/6 SAA diet would not affect fibrosis in the liver, gWAT, and iWAT (Fig. 1f; Supplementary Fig. S2a−c).

To evaluate the effects of low BCAA and SAAR diets on metabolic health, we performed glucose tolerance tests after one month on mice with these diets. Both the 1/6 BCAA and 1/6 SAA diets improved glucose sensitivity (Fig. 1g and h; Supplementary Fig. S3a). Therefore, while restriction of both BCAAs and SAAs promoted glucose tolerance, SAA restriction led to a more pronounced reduction in fat and circulating lipid concentration in both sexes. Afterward, we performed all metabolic tests in male mice. To assess whether the effects of 1/6 SAA (from here, we used SAAR diet to represent 1/6 SAA diet) are short-term or long-term, we conducted oral glucose tolerance tests (OGTTs) and intraperitoneal insulin tolerance tests for mice at earlier diet intervention timepoints (3 days and 9 days). The results showed that SAAR diet sensitized glucose and insulin responses as early as 3 days after dietary changes (Supplementary Fig. S3b and c). Given this immediate effect, we checked whether cycling the SAAR diet could maintain metabolic benefits by switching between SAAR and AA diets weekly. Consistent with the results of the short-term SAAR diet, mice lost weight after switching from the AA diet to the SAAR diet and regained it when reverting to the AA diet (Fig. 1i). Even under these alternating conditions, the fat mass remained lower, glucose sensitivity higher, and the liver and gWAT adipocyte size smaller compared to the AA control diet (Supplementary Fig. S3d−f). Additionally, we found systemic reprogramming of hormones and cytokines with SAAR diet feeding, e.g. lower insulin levels in both fasted and fed states, higher fibroblast growth factor 21 (FGF21) levels in both states which possibly contributes to higher water intake [34], decreased levels of ghrelin and GLP-1 during fasting, elevated levels of leptin, thyroxine (T4), and GLP-1 during feeding, and unchanged levels of interleukin-6 (IL-6) (Fig. 1j and k; Supplementary Fig. S4a). Notably, despite the significant fat mass loss and improved glucose tolerance with the SAAR diet, the mice behaved normally in open field tests, traveling similar distances overall and within the center area compared to mice on the AA diet (Fig. 1l). The capacity tests of the treadmill, grip, rotarod, gait, hindlimb clasping, and kyphosis all demonstrated that SAAR diet maintained the health of mice within the scope of items we examined (Supplementary Fig. S4b). Overall, the SAAR diet demonstrated better glucose sensitivity and significantly lower fat mass, alongside normal locomotion and appetite.

### SAAR diet promotes carbohydrate catabolism in the refed state for the WAT, BAT, and liver

**Figure 2:**
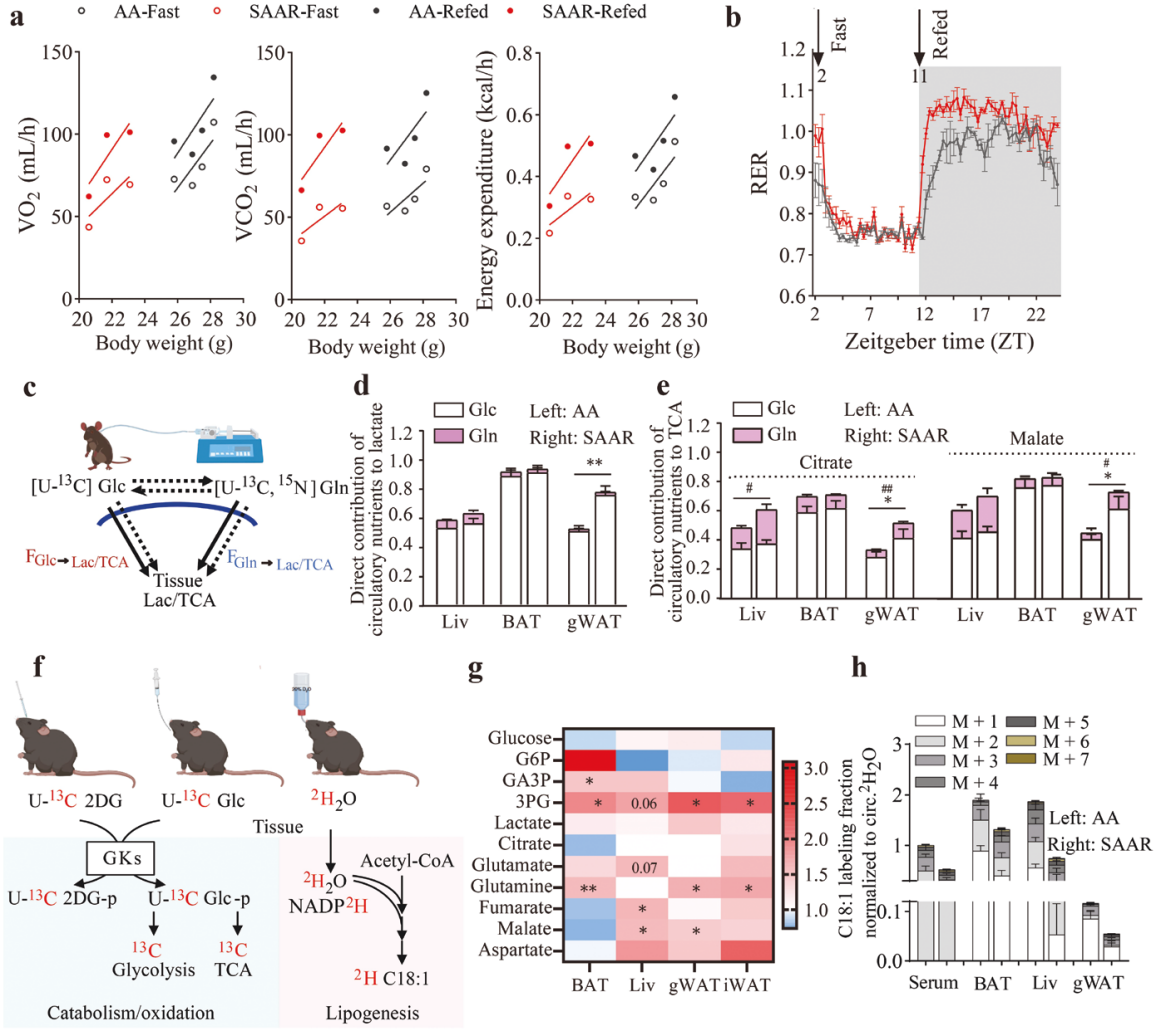
SAAR diet promotes carbohydrate catabolism in the refed state for WAT, BAT, and liver. (a) The oxygen inhale, carbon dioxide exhale rates, and EE of mice fed with AA diet and SAAR diet, normalized via ANCOVA. Oxygen consumption (VO_2_): AA_fast versus SAAR_ fast, *P* = 0.0722; AA_refed versus SAAR_refed, *P* = 0.1019; carbon dioxide production (VCO_2_): AA_fast versus SAAR_fast, *P *= 0.0614; AA_refed versus SAAR_refed, *P *= 0.0722; EE: AA_fast versus SAAR_fast, *P *= 0.1119; AA_refed versus SAAR_refed, *P *= 0.0920. Mice were fasted at 9:00 a.m. locally (Zeitgeber time 2) and 6:00 p.m. locally (Zeitgeber time 11). Mice were subjected to dietary intervention for 14 days before the experiments were performed. *n* = 4. (b) RER of mice fed with AA diet and SAAR diet from Fig. 2a. *n* = 4. (c) Scheme of *in vivo* isotope tracing of [U-^13^C] glucose and [U-^13^C,^15^N] glutamine. (d and e) Direct contribution of circulatory nutrients to lactate, citrate, and malate in different tissues (liver, BAT, and gWAT) from mice fed with AA and SAAR diets. *P* values for [U-^13^C,^15^N] glutamine tracing data are indicated with ^#^, while *P* values for [U-^13^C] glucose tracing data are marked with *. (f) Schematic diagram of [U-^13^C] 2-deoxyglucose, [U-^13^C] glucose, and ^2^H_2_O (20%) isotope tracing. (g) Fold change (SAAR/AA) of ^13^C labeling levels of metabolites in glycolysis and TCA cycle with [U-^13^C] glucose gavage. *n* = 5. (h) ^2^H labeling fraction of C18:1, normalized to circulatory ^2^H_2_O. *n* = 4. Data are expressed as mean ± SEM and statistical differences were calculated by unpaired two-tail *t*-test if unspecified. *^/#^*P* < 0.05; **^/##^*P* < 0.01; ***^/###^*P *< 0.001.

Fat loss may result from a reprogramming of oxidation and lipogenesis progress. To study changes in systemic oxidation preferences under the SAAR diet, we performed metabolic cage analysis, measuring oxygen inhalation rate (VO_2_) and carbon dioxide exhalation rate (VCO_2_) after 14 days of diet switching. Overall, these rates, including VO_2_, VCO_2,_ and EE, did not change significantly in each mouse during fasting and refeeding, even though mice with SAAR diet had significantly lower weight (Fig. 2a). The respiratory exchange ratio (RER) suggested increased carbohydrate oxidation when mice were refed with SAAR diet (Fig. 2b). To determine how organs switch nutrient utilization, we performed jugular vein catheterization and infused [U-^13^C] glucose and [U-^13^C,^15^N] glutamine into the mice for 2.5 h to reach a pseudo-steady state (Fig. 2c). The direct contributions of circulating glucose and glutamine to tissue tricarboxylic acid cycle (TCA cycle) were assessed, revealing that glucose remains the primary energy source for most organs. Increased preference for lactate production from glucose and oxidation of both glucose and glutamine were observed exclusively in gWAT (Fig. 2d and e; Supplementary Fig. S5a). To further confirm increased oxidation activity, we administered U-^13^C labeled 2-deoxyglucose with normal glucose via retro-orbital (Fig. 2f). Tissues were collected and the phosphorylation product of 2-deoxyglucose was measured via liquid chromatography-mass spectrometry (LC-MS). Most organs increased glucose intake, particularly for BAT and gWAT, with no significant change observed in the liver (Supplementary Fig. S5b). Subsequently, we gavaged mice with U-^13^C labeled glucose, finding elevated levels of its catabolic products, such as ^13^C labeled glycolytic intermediates and TCA cycle metabolites, in the liver, BAT, gWAT, and iWAT (Fig. 2g).

In addition to being catabolized into CO_2_ via the TCA cycle, nutrients also serve as precursors for lipogenesis. To illustrate the total *de novo* lipogenic flux, we provided mice with ^2^H_2_O in their drinking water for 9 days (Fig. 2f). Measurement of ^2^H enrichment in saponified lipids revealed that SAAR diet could significantly reduce ^2^H enrichment in fatty acids across the liver, BAT, gWAT, and circulating serum (using oleic acids as the representative), indicating lower *de novo* lipogenic flux compared to lipid absorbed from diet (Fig. 2h). Next, we administered ^2^H_2_O via intraperitoneal injection and collected tissue samples to calculate overnight lipid synthesis rate [28]. Measurement of the ion counts of ^2^H labeled C18:1 again suggested a lower lipid synthesis rate in the liver and WAT (Supplementary Fig. S5c). Thus, under the refed state, limiting SAA intake could promote systemic nutrient catabolism while inhibiting lipogenesis, particularly in WAT, liver, and BAT.

### SAAR diet promotes lipolysis and fatty acid oxidation in a fasting state

**Figure 3:**
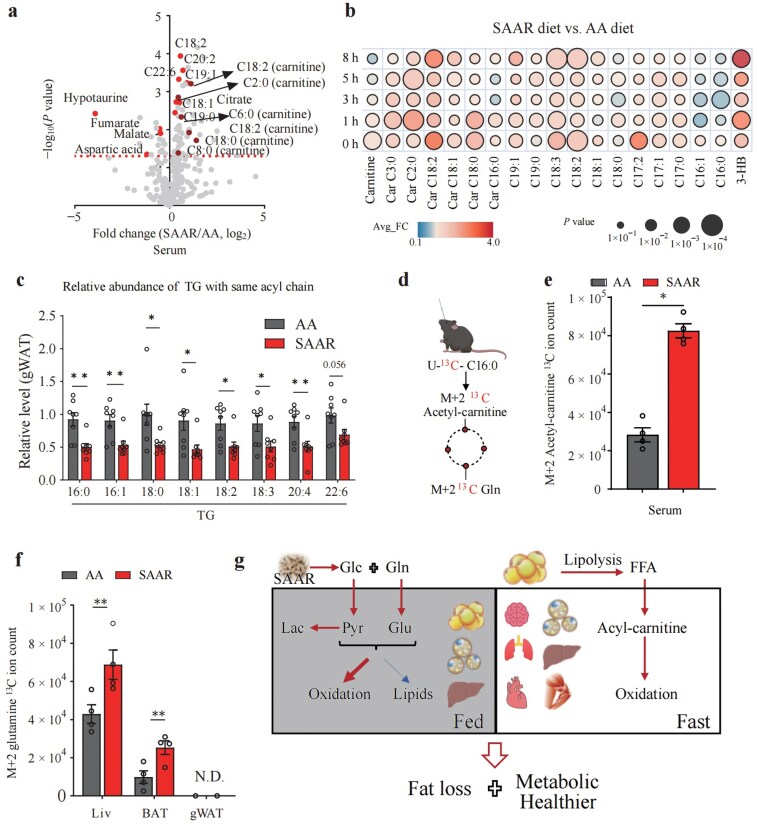
SAAR diet promotes lipolysis and fatty acid oxidation in the fasting state.(a) Volcano plot of metabolite levels of the serum from mice fed with SAAR diet compared to AA diet after 8 h of fasting. *n* = 5. (b) Fold change (SAAR/AA) of metabolites in serum from mice fed with SAAR diet and AA diet in the time course analysis of fasting. *n* = 5. (c) Relative abundance of TG with same acyl chain from lipidomic analysis of gWAT data after 8 h of fasting. (d) Schematic diagram of [U-^13^C] palmitate retro-orbital injection tracing. (e) ^13^C ion counts of M + 2 acetyl-carnitine in serum from mice fed with AA and SAAR diets. Serum was collected 30 min after retro-orbital injection of [U-^13^C] palmitic acid. *n* = 4. (f) ^13^C ion counts of tissue M + 2 glutamine for mice fed with AA and SAAR diets. Tissues were collected 30 min after retro-orbital injection of [U-^13^C] palmitic acid. *n* = 4. (g) Schematic summary of the glucose and lipid metabolism in fasting and fed states. Data are expressed as mean ± SEM and statistical differences were calculated by unpaired two-tail *t*-test if unspecified. *P < 0.05; **P < 0.01; ***P < 0.001.

Fasting can shift the body’s energy source from carbohydrate to fatty acid oxidation, with the accumulation of ketone bodies during long-term fasting [31, 35]. We performed 8 h fasting or time course fasting analysis (fasting mice for 1 h, 3 h, 5 h, and 8 h, respectively), followed by metabolic profiling of serum samples. In the serum from mice with 8 h fasting, we observed significantly elevated levels of free fatty acids resulting from lipolysis, as well as increased acyl-carnitines indicative of fatty acid oxidation (Fig. 3a). The time course fasting analysis confirmed the persistent elevation of these metabolites in the blood stream, along with an accumulation of the ketone body 3-hydroxybutyrate (Fig. 3b; Supplementary Fig. S6a and b).

We then checked metabolic changes in the liver and gWAT, as both exhibited elevated oxidation and inhibited lipogenesis upon refeeding. Gene expression analysis via bulk RNA sequencing (RNA-seq) indicated changed expression of genes related to lipid metabolism in both gWAT and liver (Supplementary Fig. S6c). Lipidomics analysis further indicated lower levels of TGs in gWAT, confirming enhanced lipolysis (Fig. 3c; Supplementary Fig. S6d). To investigate how the SAAR diet affects metabolic changes during fasting, we infused [U-^13^C] glucose and [U-^13^C,^15^N] glutamine into the mice for 2.5 h. The liver showed an increased preference for glucose and glutamine, and gWAT with increased glutamine preference, while the use of circulating glucose and glutamine in other organs was maintained (Supplementary Fig. S6e). To further understand weight loss associated with the SAAR diet at a fasting state, we injected U-^13^C labeled palmitate via retro-orbital (Fig. 3d). Higher signals of circulatory M + 2 acetyl-carnitine, a product of ^13^C palmitate oxidation, were observed (Fig. 3e). Meanwhile, most organs consumed more palmitate for TCA cycle oxidation (Fig. 3f; Supplementary Fig. 6f). The low labeling in gWAT could be attributed to its preferential use of glucose/glutamine for energy, or its storage of fatty acids as lipids, resulting in a high pool of unlabeled free fatty acids that obscures labeling. In summary, our findings indicated that the SAAR diet enhanced glucose oxidation in adipose tissues and reduced lipogenesis in the fed state, while promoting whole-body lipolysis and fatty oxidation during fasting (Fig. 3g). Consequently, fat mass got lost dramatically with limited SAA intake.

### Methionine or cystine can rescue weight loss, not due to methionine cycling

**Figure 4:**
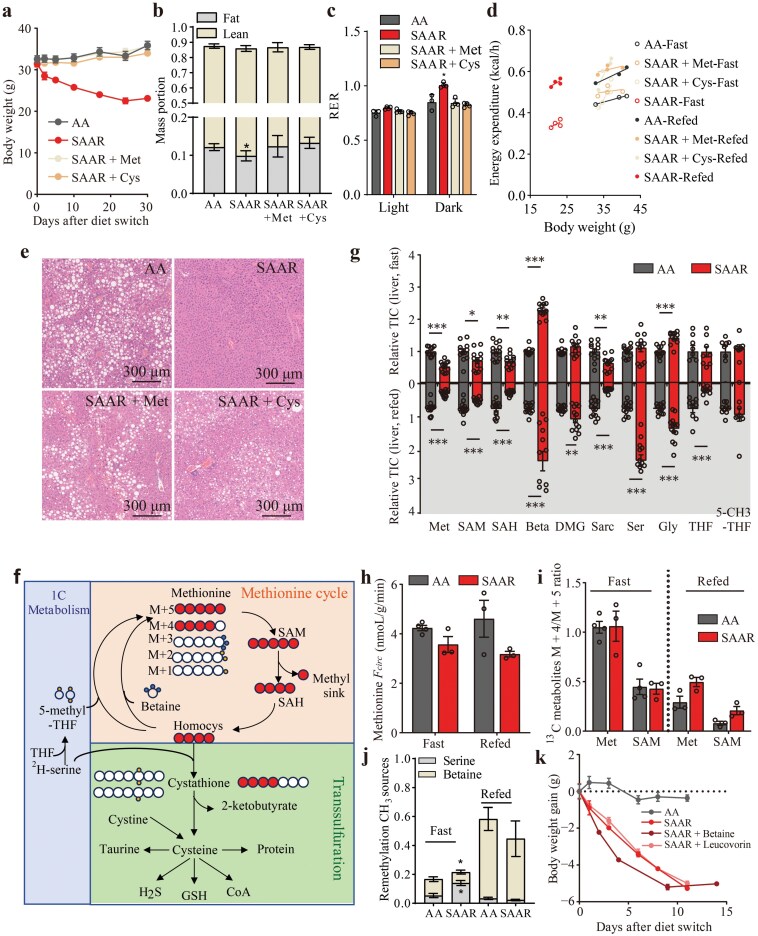
Methionine or cystine can rescue weight loss, not due to methionine cycling. (a) Weights of mice fed with different diets. AA, *n* = 4; SAAR, *n* = 5; SAAR + Met, *n* = 4; SAAR + Cys, *n* = 4. (b) Percentage of mass portions for mice fed with different diets. AA, *n* = 4; SAAR, *n* = 5; SAAR + Met, *n* = 4; SAAR + Cys, *n* = 4. (c) RER of mice fed with different diets for 40 days when the experiment was performed. *n* = 4. (d) EE of mice fed with different diets for 40 days when the experiment was performed. *n* = 4. (e) H&E staining of livers from mice fed with different diets. (f) Schematic summary of 1C metabolism, methionine cycle, and transsulfuration with [U-^13^C] methionine, [2,3,3-^2^H_3_] serine, and [trimethyl-^2^H_9_] betaine tracing. (g) Relative total ion counts (TIC) of metabolites in the livers from mice fed with AA diet and SAAR diet, normalized to AA diet. *n* = 12. (h) *F*_*circ*_ of methionine, calculated from serum tracer labeling. AA_fast, *n* = 4; AA_refed, SAAR, *n* = 3. (i) M + 4/M + 5 ratio of ^13^C ion counts of methionine and SAM in the livers from mice fed with AA diet and SAAR diet. AA_fast, *n* = 4; AA_refed, SAAR, *n* = 3. (j) Contribution of serine and betaine to CH_3_ sources of methionine remethylation in the liver. Fast, *n* = 3; refed_serine_AA, *n* = 3; refed_serine_SAAR, *n* = 4; refed_betaine, *n* = 2. (k) Weight gain for mice fed with AA diet and SAAR diet with betaine and leucovorin in drinking water or not. *n* = 4−5. Data are expressed as mean ± SEM and statistical differences were calculated by unpaired two-tail *t*-test if unspecified. *P < 0.05, **P < 0.01, ***P < 0.001.

Given that both methionine and cystine are low in the SAAR diet, we aimed to determine which of these amino acids contribute to fat loss. To achieve this, we reintroduced methionine or cystine back to the diet separately. Our findings indicated that both amino acids were effective in restoring body weight, total lean mass, and fat mass (Fig. 4a and b; Supplementary Fig. S7a and b). Metabolic cage analysis further demonstrated that the addition of methionine or cystine could rescue RER value as well as EE in each mouse with diets feeding for 30 days (Fig. 4c and d). Additionally, supplementation led to decreased sensitivity to bolus injection of glucose and insulin (Supplementary Fig. S7c). Histological staining revealed that both amino acids restored fat deposit in the liver and adipose size in gWAT (Fig. 4e; Supplementary Fig. S7d). Thus, either methionine or cystine was sufficient to reverse the previously observed benefits.

Methionine metabolism occurs through three primary pathways: the methionine cycle which converts methionine to homocysteine, a precursor for cystathionine; one-carbon (1C) metabolism, using 5-methyl tetrahydrofolate (5-methyl-THF) derived from serine or betaine as the methyl donor to regenerate methionine from homocysteine; and the transsulfuration pathway that produces cysteine and subsequently taurine, H_2_S, CoA, and other redox-related metabolites such as glutathione (GSH) (Fig. 4f). Untargeted metabolomics analysis of serum, liver, and gWAT indicated a decrease in methionine levels in the mice with SAAR diet feeding (Fig. 4g). Concurrently, metabolites in the remethylation cycle (betaine, dimethylglycine, 5-methyl-THF, serine, and glycine) increased, while metabolites in the methionine cycle (S-adenosyl methionine and S-adenosyl homocysteine) exhibited varying changes across tissues (Fig. 4g; Supplementary Fig. S7e and f). To investigate the alterations in these metabolic pathways under the SAAR diet, we infused mice with [U-^13^C] methionine, ^2^H serine, or ^2^H betaine. The turnover of methionine, as shown by the circulation flux (*F_circ_*), remained consistent in both fasting and refed states (Fig. 4h). Compared to the AA diet, *F_circ_* of serine increased with the SAAR diet, while that of betaine decreased during fasting (Supplementary Fig. S7g). Moreover, the ratios of M + 4/M + 5 methionine and S-adenosyl methionine showed no significant difference, indicating that methionine cycle flux did not decrease by the SAAR diet (Fig. 4i). Additionally, tracing with ^2^H serine or ^2^H betaine indicated that the liver preferred serine as a methyl donor for remethylation at a fasting state, however, the remethylation flux was not significantly altered by the SAAR diet (Fig. 4j). We then supplemented the drinking water with betaine or leucovorin, a folate derivative that converted into 5-methyl-THF, but found neither could prevent weight loss (Fig. 4k). These results indicate that neither the methionine cycle nor remethylation in one-carbon metabolism was the reason for fat loss.

### Cyst(e)ine, but not its downstream metabolites, controls fat loss

**Figure 5:**
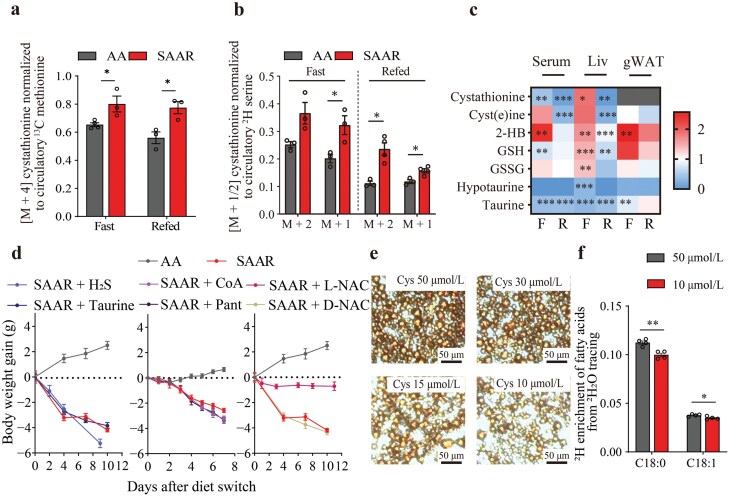
Cyst(e)ine, but not its downstream metabolites, controls fat loss. (a) Enrichment of M + 4 ^13^C labeled cystathionine in the livers from mice fed with AA diet and SAAR diet, normalized to ^13^C labeled methionine. Tissue was collected after 2.5 h of [U-^13^C] methionine tracing. Fast_AA, *n* = 4; fast_SAAR refed, *n* = 3. (b) Enrichment of M + 1 and M + 2 ^2^H labeled cystathionine in the livers from mice fed with AA diet and SAAR diet, normalized to ^2^H labeled serine. Tissue was collected after 2.5 h of [2,3,3-^2^H₃] serine tracing. Fast refed_AA, *n* = 3; refed_SAAR, *n* = 4. (c) Fold change (SAAR/AA) of metabolites in transsulfuration and downstream pathways in the serum, liver, and gWAT from mice. (d) Weight gain for mice fed with AA diet and SAAR diet, provided with GYY4137 (H_2_S), taurine, CoA, pantethine, L-NAC, and D-NAC. *n* = 4−5. (e) Oil Red O staining of SVF cells cultured in medium with different concentrations of cystine (50 μmol/L, 30 μmol/L,15 μmol/L, and 10 μmol/L). (f) ^2^H labeling of saponified fatty acid (C18:0 and C18:1) from SVF cell samples. ^2^H_2_O was added into the medium to label downstream metabolites. *n* = 4. Data are expressed as mean ± SEM and statistical differences were calculated by unpaired two-tail *t*-test if unspecified. *P < 0.05, **P < 0.01, ***P < 0.001.

Next, we examined whether cystine plays a crucial role in fat loss. Since methionine is involved in the transsulfuration pathway to produce cystathionine, a precursor for cysteine, we analyzed the labeling of cystathionine derived from [U-^13^C] methionine or ^2^H serine tracing and noticed a significant increase in labeling in response to the SAAR diet during both fasting and refed state (Fig. 5a and b). To assess whether enhanced transsulfuration could compensate for low cystine intake, we measured related metabolites in the liver, gWAT, and serum. Intracellular cysteine serves as a substrate for producing glutathione (for ROS clearance), taurine, CoA, H_2_S, and proteins (Fig. 4f). During the fasting state, the levels of cystathionine, cysteine, glutathione, and oxidized glutathione were maintained or increased. However, during the fed state, the levels of cyst(e)ine, taurine, and hypotaurine, among others, dramatically declined in both liver and serum, suggesting that enhanced transsulfuration flux could not rescue the levels of cyst(e)ine related metabolites (Fig. 5c). To investigate whether these downstream metabolites influence fat loss, we added GYY4137 (an H_2_S donor) and taurine to the drinking water or administered CoA or pantethine daily to mice with SAAR diet feeding [36, 37]. None of these interventions prevented weight loss (Fig. 5d). Since glutathione primarily functions in ROS scavenging, we added N-acetyl cysteine (NAC, both L and D forms of which could clear oxidative radicals, but only the L form can degrade into cysteine) in drinking water, and found that only L-NAC effectively rescued weight loss (Fig. 5d) [38]. In sum, cyst(e)ine, rather than their roles as precursors of taurine, H_2_S, CoA, or ROS scavenger, were key contributors to fat loss. To further validate this, we cultured immortalized adipose-derived stromal vascular fraction (SVF) cell line till lipid droplet formation. Upon switching to a medium with a low cystine level, or adding ferrostatin-1 (Fer-1), an inhibitor of ferroptosis, or a high concentration of glutathione to the medium, we found that low cystine levels decreased Oil Red O staining area, indicating reduced lipid content, while the ferroptosis inhibitor and glutathione did not reverse this effect (Fig. 5e; Supplementary Fig. S8a–e). Additionally, ^2^H_2_O tracing confirmed that low cystine levels in the culture medium inhibited *de novo* lipogenesis, reinforcing our observation that low cystine levels are linked to fat loss *in vivo* (Fig. 5f).

### The level of circulatory cystine correlates with body mass index (BMI) and total TGs

**Figure 6:**
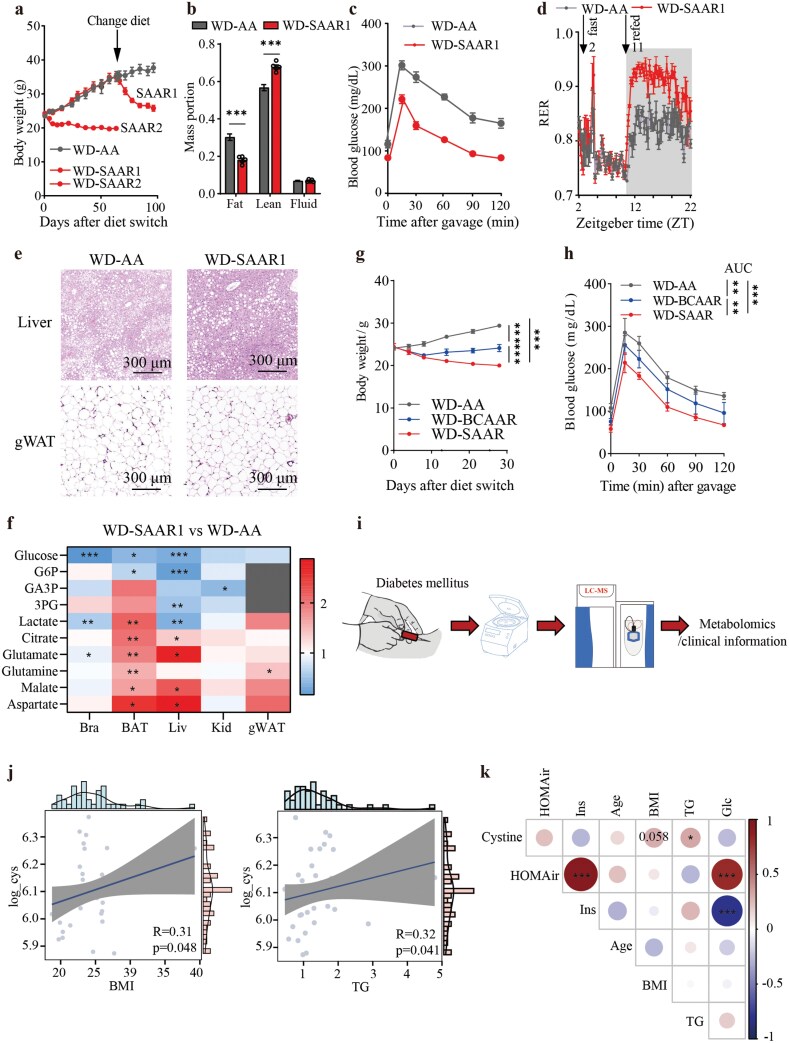
Cystine levels correlate with BMI and total TGs, suggesting its role in controlling weight in obesity. (a) Weights of mice fed with WD-AA diet, WD-SAAR1, or WD-SAAR2 diet. WD-SAAR1 is a diet intervention that switches diets from the WD-AA diet after 60 days, and WD-SAAR2 is a diet intervention that switches diets at day 1. *n* = 5. (b) Percentage of mass portions for mice fed with WD-AA diet and WD-SAAR1 diet. *n* = 5. (c) Circulatory glucose levels in mice fed with WD-AA diet and WD-SAAR1 diet gavaged with 1 g/kg glucose after 16 h of fasting. *n* = 5. (d) RER of mice fed with WD-AA diet and WD-SAAR1 diet. Experiments were performed after mice were fed either with a WD-SAAR diet for 25 days after 60 days of WD-AA feeding, or continuous 85 days with WD-AA feeding. *n* = 4. (e) H&E staining of livers and gWAT from mice fed with WD-AA diet and WD-SAAR1 diet. (f) Fold change (WD-SAAR1/WD-AA) of ^13^C TCA cycle metabolites in mice gavaged with 1 g/kg U-^13^C glucose. (g) Weights of mice fed with WD-AA, WD-SAAR, or WD-BCAAR diet. *n* = 5. (h) Circulatory glucose levels in mice fed with WD-AA, WD-SAAR, or WD-BCAAR diet gavaged with 1 g/kg glucose after 16 h of fasting. *n* = 5. (i) Flowchart of serum metabolite analysis in diabetic patients. (j) Spearman correlation analysis between serum cystine levels and BMI or total TGs. (k) Partial Spearman correlation analysis between serum cystine concentrations and obesity-related indicators in the population. Data are expressed as mean ± SEM and statistical differences were calculated by unpaired two-tail *t*-test if unspecified. *P < 0.05, **P < 0.01, ***P < 0.001.

To translate our findings to disease models, we formulated Western diets (WD), in which proteins were replaced with either equivalent amino acids or low SAAs (Supplementary Table S2). Switching to these diets on the first day or after more than two months of WD feeding till weight reached a steady state, significantly reduced body weight, particularly fat mass (Fig. 6a and b; Supplementary Fig. S9a). Glucose tolerance tests and metabolic cage assessments confirmed that SAAR diet enhanced glucose tolerance and shifted metabolism toward carbohydrate burning in mice with WD (Fig. 6c and d; Supplementary Fig. S9b−d). We also observed loss of lipid droplets in the liver and a reduced size of adipose in the gWAT (Fig. 6e; Supplementary Fig. S9e). To further validate enhanced carbohydrate oxidation, we gavaged [U-^13^C] glucose to the mice, finding significantly higher oxidation in the gWAT, BAT, and liver (Fig. 6f; Supplementary Fig. S9f). A direct comparison of mouse weight and glucose tolerance further corroborated that SAAR diet performed better than a low BCAA diet, even in a westernized diet (Fig. 6g and h). For clinical relevance, we measured cystine levels in plasma drawn from diabetic patients (Fig. 6i; Supplementary Fig. S9g). Spearman correlation analysis revealed that circulating cystine levels were strongly correlated with BMI and total TG levels, highlighting cystine as a potential regulator of obesity in diabetic patients, consistent with discoveries from other human studies in non-diabetic obese patients (Fig. 6j and k) [39–41].

## Discussion

Our studies demonstrated that SAAs, particularly cystine, play a crucial role in regulating systemic glucose and lipid homeostasis, as proven in cystathionine γ-lyase deficient mice fed with a cystine-free diet [42]. Similar to existing studies which found that cystine level correlates with BMI in non-diabetic obese patients and SAAR diet could be a beneficial dietary strategy for weight loss, our discovery proved the potential role of cystine in controlling weight for diabetic patients [39–41, 43, 44]. However, unlike previous studies that focused on the expression changes of gene, protein, or cytokine, here we employed metabolomics and *in vivo* isotope tracing to quantify systemic and organ-level fluxes in the TCA cycle and *de novo* lipogenesis. SAAR diet promotes fat loss through mechanisms such as inhibiting *de novo* lipogenesis, redirecting carbohydrates and amino acids into TCA oxidation, releasing CO_2_ during feeding, and enhancing lipolysis and fatty acid oxidation during fasting. This disruption in lipid balance leads to rapid fat loss. Using *in vivo* isotope tracing, we found that the gWAT, BAT, and liver had reduced lipid synthesis fluxes, while the heart, brain, BAT, and lungs showed increased fatty acid burning. Expression levels of thermogenic genes in iWAT suggested the beigeing of WAT, correlating to high catabolic metabolism for adipose tissue with the SAAR diet (Supplementary Fig. S10a; Supplementary Table S3). *Ex vivo* differentiation of SVF cells confirmed that cystine regulates lipid droplet formation, essential for fat storage.

To explore the molecular mechanism, we probed the activities of AMP-activated protein kinase (AMPK), the mechanistic target of rapamycin (mTOR), activating transcription factor 4 (ATF4), eukaryotic translation initiator factor 2α (elf2α), and protein kinase RNA-like ER kinase (PERK) by measuring their total and phosphorylation levels in the liver and gWAT. We found that SAAR diet could only increase ATF4 levels in gWAT, similar to BCAA restriction (BCAAR) diet, and AMPK phosphorylation in the liver (Supplementary Fig. S10b; Supplementary Table S3). However, SAAR diet could not affect the activities of others in both gWAT and the liver with 30 days of SAAR diet. The different results between our study and others could be due to the number of days after diet switching or whether samples were collected at fasting or refed state [45]. Even though multiple genetic models were utilized to prove the molecular mechanisms of the benefits brought by diet manipulation, it remains elusive and challenging [20, 32, 33, 46]. One possible reason is that these benefits arise from multi-organ interactions and feedback loops. For example, low protein diets or low BCAA diets are well-known strategies to enhance glucose sensitivity and longevity [16, 17, 47]. It is also established that restricting specific BCAAs, particularly valine and isoleucine, improves metabolic benefits, while leucine deprivation alone can reduce food intake and fat mass via the general control nonderepressible 2 (GCN2) signaling in neurons [20, 48, 49]. Explorations using genetic models, such as conditional knockout of branched-chain α-ketoacid dehydrogenase (BCKDH), an enzyme catabolizing BCAA oxidation, have yielded inconsistent results compared to low-BCAA diet or BCKDH kinase inhibitors like 3,6-dichlorobenzo(b)thiophene-2-carboxylic acid (BT2) [21, 50]. Similarly, manipulating the expression of key genes like FGF21, mTOR, and GCN2, has not fully replicated the phenotypes observed in these nutrient interventions [20, 32, 33]. These insights suggest that different nutrient restrictions result in varying metabolic outcomes, making it difficult to pinpoint a single mechanism. It highlights the need for a deeper understanding of nutrient processing, such as sensing, absorption, transport, and metabolism, and how these processes interact across multiple organ systems, as well as with the microbiota [51].

In our study, we identified WAT, BAT, and liver as key organs responsible for fat loss during the SAAR diet and observed changes in hormones such as insulin, FGF21, leptin, thyroid hormone (T4), and GLP-1. Further investigation is required to apply these findings clinically. To distinguish the effects of methionine and cystine on whole-body metabolism, we traced methionine flux in the liver, the main site of the methionine cycle, including processes such as S-adenosylmethionine (SAM) synthesis for methylation and phosphocholine biosynthesis. Although methionine fluxes in the liver did not decrease, methionine restriction may enhance the outcomes of cystine deficiency. For instance, we observed improved insulin sensitivity when cystine was sufficient while methionine was relatively low in the diet (Supplementary Fig. S7c). Additionally, we did not explore methylation fluxes for each fate in depth within the SAAR diet context. To extend our research, we could generate cystathionine β-synthase-deficient mice to further investigate the role of cystine [52].

Furthermore, recent studies have made nutrient intervention even more exciting. For example, taurine and acetyl-taurine, the major cysteine-derived products, have been shown to reduce abdominal fat and extend lifespan across species [53, 54]. However, in our study, taurine in drinking water could not replicate cystine’s effects, indicating that other metabolic pathways may be responsible for fat loss. Thus, to study the mechanism of fat loss, a comprehensive exploration of the functions of small molecules derived from cyst(e)ine, or the roles of specific proteins requiring abundant cysteine as substrate, which we have not touched in our study, is needed, but challenging and worthy to explore for chronic metabolic syndromes, like obesity here.

Last but not least, health concerns arise since the requirements of nutrients across organs are so different from each other, even though diet manipulations have been proven beneficial for multiple metabolic diseases. It has been shown that a low protein diet could lead to muscle loss, and a low carbohydrate diet could cause side effects like constipation, headache, and muscle cramps [55–58]. In our study, we measured organ weight and found that SAAR diet majorly affects fat in tissues since muscle mass was maintained (Supplementary Fig. S1e). Additional measurement of the relative rates of essential amino acid incorporation into new protein in the liver and pancreas or its circulatory turnover during the fasting state with [U-^13^C] methionine tracing confirmed that SAAR diet did not affect protein metabolism (Supplementary Fig. S10c). Furthermore, we measured mouse behaviors like activities of treadmill, grip, rotarod, gait, hindlimb clasping, and kyphosis, and found that mice in the SAAR diet maintained healthy. Other issues related to health are still required to be monitored closely if a long-term SAAR diet is executed.

## Limitations of the study

Several limitations were observed in this study. First, our study utilized diets with ultra-low levels of both methionine and cystine to induce weight loss, increased insulin sensitivity, and reprogrammed glucose-lipid metabolism across organs. It is complex to dissect the contribution of each SAA to the aforementioned phenotypes, even though we found that a low level of cystine majorly leads to fat loss. Mice deficient in the activity of cystathionine γ-lyase or cystathionine β-synthase are helpful to explore further. Second, we found similar EE for mice fed with SAAR and AA diets, even though weight in the SAAR group was much lower than that in the group of AA diet. However, regression-based analysis of covariance (ANCOVA) using body mass as a covariate only showed a trend toward higher mass-independent metabolic rates in the SAAR group for insufficient replicates. Third, we also did not definitively determine whether a long-term SAAR diet could lead to health issues, since we only checked behaviors like activities of treadmill, grip, rotarod, gait, hindlimb clasping, and kyphosis for mice with 30 days of SAAR diet feeding. It remains unaddressed whether the SAAR diet is applicable for all models aiming for weight loss. The benefits or potential harms of low SAA diets on human beings were also not determined clinically. Furthermore, we found that downstream metabolites of cyst(e)ine could not rescue fat loss, and the SAAR diet did not affect the activities of AMPK, mTOR, elf2α, and PERK in gWAT. In this paper, we did not explore further the mechanisms of cyst(e)ine in controlling lipolysis. In conclusion, our study shows that SAAR diet could result in weight loss for mice in normal chow and high-fat diets. More experiments are required to investigate the molecular mechanisms and their potential applications in clinics.

## Materials and methods

### Mouse studies

All mice were on the C57BL/6J genetic background. Mice were maintained in a specific pathogen-free facility and kept on a 12-h light/12-h dark cycle with free access to food and water. All animal experimental protocols were approved by the Institutional Animal Care and Use Committee Institutional Animal Care and Use Committee of Shanghai Institute of Nutrition and Health, Chinese Academy of Sciences with an approval number SINH-2023-YLF-1. For sample collection, mice were fasted for 8 h from 9:00 a.m. to 5:00 p.m. (fasting) or fasted from 10:00 a.m. to 6:00 p.m. and refed for 2 h for the refed state. For isotope tracing test, mice were fed for 7−9 days before tracing and sacrificed if unspecified. For the test of small molecules derived from cysteine (Fig. 5), GYY4137 (S9920, Selleck), Taurine (107-35-7, Psaitong), N-acetyl-D-cysteine (14128926, adamas), and N-acetyl-L-cysteine (HY-B0215, MCE) were prepared in drinking water, and pantethine (HY-B1028, MCE) and CoA hydrate (ST353-200 mg, Byotime) were prepared in saline for retro-orbital venous injection.

### Diets

The diets used in this study included the AA diet (FB-A10022B AA), 1/3 BCAA diet (FB-A10022B 1/3 BCAA), 1/6 BCAA diet (FB-A10022B 1/6 BCAA), 1/3 SAA diet (FB-A10022B 1/3 SAA), 1/6 SAA diet (SAAR; FB-A10022B 1/6 SAA), SAAR + Cys diet (FB-A10022B SAAR + Cys), SAAR + Met diet (FB-A10022B SAAR + Met), Western-AA diet (FB-WD-AA), Western-SAAR diet (FB-WD-SAAR), and Western-BCAAR diet (FB-WD-BCAAR) (Supplementary Tables S1 and S2). The AA diet was created by replacing the casein in a standard chow diet with a mixture of amino acids that matched the amino acid profile of casein. For the 1/3 BCAA and 1/6 BCAA diets, as well as the 1/3 SAA and 1/6 SAA diets, the amino acid profiles were modified by decreasing the levels of BCAAs (leucine, isoleucine, and valine) and sulfur-containing amino acids (SAAs: methionine and cysteine) to 1/3 and 1/6 of their original concentrations in the AA diet (notes: cystine is removed from 1/3 SAA and 1/6 SAA diet). The SAAR + Cys and SAAR + Met diets were developed by supplementing methionine and cysteine to the levels present in the AA diet, based on the 1/6 SAA diet. The WD-AA diet was formulated by substituting the protein in a typical Western diet with an equivalent amino acid mixture, while the WD-SAAR diet involved reducing the levels of methionine to 1/6 and cysteine to 0%, and the WD-BCAAR diet involved reducing the levels of BCAAs to 1/6 of their original level within the framework of the WD-AA diet.

### Body composition and metabolic cage analyses

The physical composition of the mice was evaluated using the EchoMRI system (Houston). Following the guidelines provided by the manufacturer, measurements of the total fat mass and lean mass were obtained for each individual mouse. In conducting the metabolic cage analysis, mice were selected at random to determine their metabolic rates with the comprehensive laboratory animal monitoring system (CLAMS-16, Columbus Instruments), adhering to the manufacturer’s protocols. The weights of the mice were recorded, and they were allowed 24 h to acclimate to the system before data collection commenced. Subsequently, data were gathered over a 48-h period. During this period, the VO_2_, VCO_2_, and RER were monitored. All data points were collected at 16-min intervals. The designated time points for fasting and refeeding were 9:00 a.m. locally (Zeitgeber time 2) and 6:00 p.m. locally (Zeitgeber time 11), respectively. Data analysis was performed via CalR.

### Glucose tolerance test and insulin tolerance test

For the glucose tolerance test, the mice were fasted overnight for 16 h and subsequently given glucose solution (1 g/kg; Psaitong) by gavage. For insulin tolerance test, the mice were fasted for 4 h from 9 a.m. and subsequently injected intraperitoneally with insulin solution (1 U/kg; Novo Nordisk). Blood glucose was determined from the tail vein at 0, 15, 30, 60, 90, and 120 min after glucose or insulin administration using a glucometer (Bayer).

### Differentiation of SVF and cell culture

SVF cells were cultured in Dulbecco’s Modified Eagle Medium (DMEM, C12430500BT, ThermoFisher) with 100 units/mL penicillin/streptomycin (MA0110, Meilunbio) and 10% fetal bovine serum (FBS, F8318-500ML, Sigma) at 37°C with 5% CO_2_. For SVF differentiation, confluent SVF cells were cultured with DMEM with FBS for 2 days and then incubated with 0.5 mmol/L isobutylmethylxanthine (IBMX, HY-12318, MCE), 1 μmol/L dexamethasone (HY-14648, MCE), 20 nmol/L rosiglitazone (HY-17386, MCE), and 10 μg/mL insulin (I10022, Psaitong) for 2 days. The medium was then replaced every 1 or 2 days with DMEM containing 10 μg/mL insulin for 6−8 days, and these cells were ready for other tests.

For experiments of different cystine concentrations, medium was formulated by adding 4 mmol/L glutamine, 50 μmol/L methionine, and 10, 15, 30, 50 μmol/L cystine, using DMEM (without glutamine, methionine, and cystine, 21013024 Thermofisher). After the completion of SVF maturation, the medium was changed daily for a total of 4 days. Then, SVF cells were stained with Oil Red O (C0158S, Byotime).

For the Fer-1 test and glutathione test, we added 8 μmol/L Fer-1 (HY-100579, MCE) or 200 μmol/L glutathione into the medium with 10 μmol/L cystine. Oil Red O staining was performed after 3 days of treatment.

### Sample preparation and metabolite extraction for LC-MS

Serum samples were collected from tail vein, following with centrifuging at 14,000 rpm at 4°C for 10 min. Serum was collected and stored at −80°C till extraction. Three microliters of serum were mixed with 120 μL of 100% methanol, vortexed sufficiently, and centrifuged twice at 14000 rpm for 30 min each at 4°C. Then, the supernatant was used for LC-MS analysis.

Tissue samples were clamped by Wollenberger clamp into liquid nitrogen immediately after cervical dislocation of mice. Samples were stored at −80°C till extraction. Frozen tissues were pulverized using a Cryomill (Retsch). The resulting powder (10−20 mg) was weighed into a precooled tube and mixed well with 40% of methanol, 40% of acetonitrile, and 20% of water containing 0.5% of formic acid (40 μL of solvent per mg tissue) at −20°C. After sufficient vortex, 15% of NH_4_HCO_3_ solution [8.5% (v/v) of extraction buffer] was added to neutralize formic acid. Samples were centrifuged twice for 30 min each at 4°C, and the supernatant was transferred for LC-MS analysis.

### Saponified fatty acid extraction

The extraction of saponified fatty acids from tissues involved the addition of 1 mL of 0.3 mol/L potassium hydroxide in a 90:10 methanol/water solution to the powdered tissue sample. The mixture was then transferred to a 4-mL glass vial and subjected to saponification at 80°C in a water bath for a duration of 1 h. Following saponification, the samples were allowed to cool to ambient temperature, neutralized with 100 μL of pure formic acid, and subsequently extracted with 1 mL of hexane. The hexane layer was decanted into a separate glass vial, evaporated to dryness under nitrogen, and then reconstituted in a 1:1 mixture of methanol and acetonitrile (100 μL/mg tissue; 40 μL/μL serum; 200 μL/million cells) for subsequent LC-MS analysis.

### Sample preparation for folate measurement

The quantification of folate species was also conducted using a derivatization technique. Initially, 20 mg of tissue was subjected to extraction with 1 mL of a 1:1 mixture of methanol and water, supplemented with sodium ascorbate (25 mmol/L) and ammonium acetate (25 mmol/L). The resulting precipitate was separated by centrifugation at 16,000 *g* for 10 min. The supernatants were then evaporated to dryness under nitrogen and reconstituted in 450 μL of water containing ascorbic acid (30 mmol/L), dipotassium phosphate (50 mmol/L), and 2-mercaptoethanol (0.5%). Subsequently, 25 μL of charcoal-treated rat serum was mixed with each sample, followed by an incubation period of 2 h at 37°C. Sample cleanup was performed using Bond Elut PH columns (Agilent Technologies). The columns were prepped with 1 mL methanol and equilibrated with 1 mL ascorbic acid buffer (30 mmol/L ascorbic acid, 25 mmol/L ammonium acetate in water). The samples were adjusted to a pH of 4 with formic acid before being loaded onto the columns. Each column was then rinsed with 1 mL ascorbic acid buffer. The folate species were eluted using 400 μL of a 1:1 mixture of methanol and water, containing 2-mercaptoethanol (0.5% vol/vol) and ammonium acetate (25 mmol/L). The eluate was dried under nitrogen, reconstituted in HPLC-grade water, centrifuged at 16,000 *g* for 5 min, and finally analyzed by LC-MS.

### LC-MS method

For metabolomics analysis and the detection of isotopically labeled products resulting from ^13^C tracing, a Q-Exactive Plus Orbitrap mass spectrometer coupled with a Vanquish Ultra Performance Liquid Chromatography system (both from Thermo Fisher Scientific) was utilized. The Hilic column (2.1 mm × 150 mm, 5 μm; HILICON) was kept at a constant temperature of 25°C throughout the analysis. The mobile phase consisted of solvent A, which was 95% water, 5% acetonitrile, 20 mmol/L ammonium acetate, and 20 mmol/L ammonium hydroxide at a pH of 9.4, and solvent B, which was acetonitrile. The gradient program was as follows: 0−2 min, 95% B; 3−7 min, 75% B; 8−9 min, 70% B; 10−12 min, 50% B; 13−14 min, 25% B; 16−20.5 min, 0% B; and 21−28 min, 90% B. The sample injection volume was 5 μL. The Q-Exactive Plus mass spectrometer was set to operate in a switching negative/positive ion mode, scanning the mass-to-charge ratio (*m/z*) range from 60 to 900 with a resolution of 140,000 at *m/z* 200 (AGC target 3e6, Maximum IT 200 ms). The LC-MS data were collected and analyzed using El-Maven software, and subsequent natural abundance correction was performed with isoCor.

### Folate detection by LC-MS

For folate analysis, the Q-Exactive PLUS quadrupole-orbitrap MS was employed with an Agilent Poroshell 120 Bonus-RP column (2.7 μm, 2.1 mm × 150 mm). The LC gradient used solvents A (1% 1 mol/L ammonium acetate and 0.1% glacial acetic acid) and B (acetonitrile): 4 min, 80% B; 10 min, 2% B; 6 min, 30% B; 11 min, 100% B; 15 min, 100% B; 16 min, 2% B; 20 min, 2% B. The flow rate was 200 μL/min at 25°C, with 20 μL injection. MS scans were in negative-ion mode, 35,000 resolutions at *m/z* 200, and 350−1,000 *m/z* range, with an AGC target of 1 × 10^6^.

### Fatty acid detection by LC-MS

For fatty acid analysis, an Exactive Orbitrap MS was used with LC separation on a Luna C18 column (150 mm × 2.0 mm, 3 μm, 100 Å) using a gradient of solvent A (10 mmol/L tributylamine, 15 mmol/L acetic acid in 97:3 H_2_O:methanol, pH 4.5) and solvent B (methanol): 0 min 80% B; 10 min 90% B; 11 min 99% B; 25 min 99% B; 26 min 80% B; 30 min 80% B. The flow rate was 250 μL/min at 25°C, with 5 μL injection. MS scans were in negative-ion mode, 100,000 resolutions at *m/z* 200, 120−600 *m/z* range, with high dynamic range AGC target.

### *In vivo* [U-^13^C]-glucose gavage

Mice were fasted from 9:00 a.m. to 5:00 p.m., and blood samples (~20 μL) were collected by tail bleeding at 0 min time point. Then, mice were gavaged with water containing [U-^13^C] glucose (1 mg/g, body weight (10 μL/g); CLM-1396-PK, Cambridge Isotope Laboratories). After 40 min, blood samples (~20 μL) were collected from tail vein, and mice were euthanized by cervical dislocation. Tissue samples were clamped for sample preparation and analysis.

### *In vivo* isotope infusion

Mouse jugular vein catheter surgery was performed 1 week before infusion for [U-^13^C] glucose, [2,3,3-D₃] serine (DLM-1073, Cambridge Isotope Laboratories), [U-^13^C] methionine (CLM-893-H, Cambridge Isotope Laboratories), [trimethyl-D_9_] betaine (sigma, 616656), and [U-^13^C, ^15^N] glutamine (CLM-1822-H, Cambridge Isotope Laboratories) tracing. The infusion was performed for 2.5 h to achieve isotopic pseudo-steady state. The infusion setup included a tether and swivel system, connecting to the button pre-implanted under the back skin of mice. For the fasting state, mice were fasted from 9:00 a.m. to 2:30 p.m. and infused from 2:30 p.m. till 5:00 p.m. For the refed state, mice were fasted from 10:00 a.m. to 6:00 p.m. and refed for 1 h, and then infused 2.5 h, with food in the cage. Tracers were all dissolved in saline. The concentrations of infusion were [U-^13^C] glucose at 500 mmol/L for refed state, [2,3,3-D₃] serine at 400 mmol/L for fasting state and 400 mmol/L for refed state, [U-^13^C] methionine at 15 mmol/L for fasting state and 30 mmol/L for refed state, [trimethyl-D_9_] betaine at 10 mmol/L for fasting state and 20 mmol/L for refed state, and [U-^13^C, ^15^N] glutamine at 150 mmol/L for refed state. Infusates were infused via the catheter at a constant rate (0.1 μL/min per g mouse weight) using a syringe pump. At the end of infusion, mice were euthanized by cervical dislocation. Blood and tissue samples were then collected.

### *In vivo* isotope retro-orbital injection

Mice were fasted overnight from 6:00 p.m. (day 0) to 9:00 a.m. (day 1) and were sacrificed 40 min after a retro-orbital injection for the 2-deoxyglucose (2-DG) test. [U-^13^C] 2-deoxy-D-glucose (CLM-10466-PK, Cambridge Isotope Laboratories) and normal glucose were dissolved in saline solution at a concentration ratio of 1:9 (1 mg/g).

Mice were fasted from 9:00 a.m. to 5:00 p.m. and were subsequently sacrificed 40 min after a retro-orbital injection for the palmitate test. For the preparation of [U-^13^C] palmitic acid (CLM-409-0.1, Cambridge Isotope Laboratories) infusates, tracers were formulated as a BSA-conjugated solution with a final concentration of 10 mmol/L. Specifically, a 2.5 mmol/L BSA solution was prepared by dissolving BSA in saline at 37°C, and 25 mmol/L palmitic acid solution was dissolved in saline at 70°C. Maintaining the BSA solution at 37°C, the 70°C palmitic acid solution was incrementally added to the BSA solution, with sufficient vortexing performed after each addition of palmitic acid until complete dissolution was achieved.

Mice were injected with 2-deoxy-D-glucose mix solution or BSA-conjugated palmitic acid solution at a dosage of 5 μL/g.

### *In vitro* and *in vivo*^2^H_2_O tracing

For ^2^H_2_O drinking water test, mice were provided with 20% ^2^H_2_O (DLM-4-1L, Cambridge Isotope Laboratories) in drinking water for 9 days. Serum and tissue samples were collected for LC-MS analysis.

For ^2^H_2_O intraperitoneal injection test, mice were injected intraperitoneally with 30 μL/g ^2^H_2_O (in 0.9% NaCl) at 8:00 p.m. Following the injection, mice were provided with 6% deuterium water to maintain enrichment throughout the experiment. At 10:00 a.m. of the next day, serum and tissue samples were collected for LC-MS analysis.

For the ^2^H_2_O tracing of SVF cell lines, 20% ^2^H_2_O medium were prepared with different concentrations of cystine. Samples were collected after 3 days of tracing.

### Histology and data analysis

Freshly collected liver, gWAT, and iWAT tissues from mice were fixed in 4% paraformaldehyde, embedded in paraffin, sectioned, and stained with hematoxylin and eosin (H&E) or Masson. The processing and histopathology reporting were performed by Wuhan Servicebio Technology Laboratory and Hubei Biossci Technology Laboratory.

For SVF cell Oil Red O staining, cells were treated with the Oil Red O Kit (C0158S, Beyotime). The area of the stained lipid droplets was quantified using ImageJ.

### Rotarod test

All behavior tests were performed after SAAR diet feeding for 30 days. For the rotarod test, the mice were positioned on a rotarod cylinder (XR-6C, Xinruan) that progressively accelerated, and their latency time to fall was recorded. The rotation speed was increased from 4 to 40 rpm within 5 min. The trial was ended when the mouse either fell from the cylinder or clung to it and spun around twice in a row without trying to walk. The results from the motor skill test were reported as the best latency time of three attempts for the mice on the rotarod.

### Grip strength test

The grip strength of the mice was evaluated using a grip strength testing machine (XR501, Xinruan). Each mouse was tested three times to determine its performance. For the experiment, the mice were made to hold onto a metal grille using their front limbs, or a combination of their front and rear limbs. The mouse’s tail was softly tugged, and the maximum force exerted was recorded by the force sensor when the mouse released its grasp on the grille. The highest grip force was captured digitally and presented as the force measured in gram force (gf). The grip strength results were reported as the best maximum force of three trials.

### Treadmill test

The muscle endurance of mice was measured using a treadmill (XR-PT-10B, Xinruan). The electric shock of the treadmill was set to 0.4 mA at 3 Hz, and the mouse was considered exhausted when shocked continuously for 5 s. At a slope angle of 10°C, the treadmill speed was maintained at 10 m/min for 5 min, then accelerated by 1 m/min^2^ until the mouse became exhausted. The exhaustion time for each mouse was recorded.

### Quantitative PCR (qPCR) analysis

Total RNA was isolated from tissue samples using the FastPure Complex Tissue/Cell Total RNA Isolation Kit (RC113-01, Vazyme) according to the manufacturer’s protocol. RNA concentration and purity were assessed using a Nanodrop spectrophotometer (Thermo Scientific). Complementary DNA (cDNA) was synthesized from 1 μg RNA using the iScript cDNA Synthesis Kit (Bio-Rad) following the manufacturer’s instructions. qPCR was performed using the SYBR Green Supermix (Bio-Rad) on a CFX96 Real-Time PCR System (Bio-Rad). Gene expression was quantified using the ΔΔCt method, and expression levels were normalized to *β-Actin* as the housekeeping gene. Amplification conditions were as follows: an initial denaturation at 95°C for 3 min, followed by 40 cycles of denaturation at 95°C for 15 s, annealing at 60°C for 30 s, and extension at 72°C for 30 s. Melt curve analysis was performed to verify primer specificity. Data were analyzed using the Bio-Rad CFX Manager software, and relative expression was calculated using the 2^*−*^^ΔΔCt^ method. Primers for qPCR were deposited in Supplementary Table S3.

### Western blot analysis

Tissue samples were homogenized in RIPA lysis buffer (Meilun Bio) containing protease and phosphatase inhibitors (Beyotime). Protein concentrations were determined using the BCA Protein Assay Kit (Meilun Bio). Equal amounts of protein (30−50 μg) were separated by SDS-PAGE (10% gel) and transferred to PVDF membranes (Millipore). The membranes were blocked with 5% BSA in TBS-T buffer for 1 h at room temperature and incubated overnight at 4°C with primary antibodies specific to the proteins of interest. After washing with TBS-T buffer, membranes were incubated with appropriate HRP-conjugated secondary antibodies (1:2000 dilution) for 1 h at room temperature. Protein bands were visualized using SuperSignal West Pico Chemiluminescent Substrate (Meilun Bio) and detected using a ChemiDoc MP Imaging System (Tanon). Densitometric analysis was performed using ImageJ software, and protein expression levels were normalized to HSP90. Data are presented as relative protein expression. Antibody information was deposited in Supplementary Table S3.

### Insulin, leptin, T4, ghrelin, GLP-1, FGF21, IL-6, TG, TC, and ALT assays

Serum samples were collected from the left ventricle during anesthesia or via the tail vein. The levels of insulin, leptin, T4, ghrelin, and GLP-1 in serum were measured using the following kits: Mouse Insulin ELISA Kit (MS100, EZassay), Mouse Leptin ELISA Kit (MM-0622M2, Jiangsu Meimian Industrial Co., Ltd), Mouse T4 ELISA Kit (MM-0575M2, Jiangsu Meimian Industrial Co., Ltd), Mouse Ghrelin ELISA Kit (MM-0621M2, Jiangsu Meimian Industrial Co., Ltd), Mouse GLP-1 ELISA Kit (MM-0027M2, Jiangsu Meimian Industrial Co., Ltd), Mouse FGF21 ELISA Kit (MM-44032M2, Jiangsu Meimian Industrial Co., Ltd), Mouse Homocysteine ELISA Kit (MM-0294M2, Jiangsu Meimian Industrial Co., Ltd), and Mouse IL-6 ELISA Kit (MM-0163M2, Jiangsu Meimian Industrial Co., Ltd). Hepatic TG, TC, and ALT levels were assessed using the Cholesterol Assay Kit (A111-1-1), Triglyceride Assay Kit (A110-1-1), and Alanine Aminotransferase (ALT) Assay Kit (C009-2-1), all of which were obtained from Nanjing Jiancheng Bioengineering Institute and analyzed according to the manufacturer’s instructions.

### Lipidomics analysis

Lipid extraction was conducted utilizing a modified methyl tert-butyl ether (MTBE) method. Tissue (10−50 mg) was homogenized in 200 μL water, employing a cooled nitrogen gas flow from liquid nitrogen for three cycles (each cycle: 5500 rpm for 20 s, repeated three times). MTBE (400 μL) and methanol (MeOH, 80 μL) were added to 200 μL of plasma/serum/tissue extract. The samples were vortexed for 30 s and then sonicated for 10 min at 4°C. Then the samples were centrifuged at 3,000 rpm for 15 min to separate phases (white precipitate will appear at the interface). The upper MTBE phase was carefully transferred to a new Eppendorf tube and dried under a gentle stream of nitrogen gas until completely dry. The dried lipid extracts were then reconstituted in 100 μL of a 1:1 (v/v) mixture of dichloromethane (DCM) and MeOH. A 5-μL aliquot of each sample was used as a pooled quality control (QC) sample, which was analyzed once every 8 samples to ensure consistency and accuracy throughout the experiment. Samples were then centrifuged for 30 min at 14,000 rpm, and the supernatant was used for LC-MS analysis.

### RNA isolation and library preparation

Total RNA was extracted utilizing the TRIzol reagent (Invitrogen, CA, USA) in accordance with the manufacturer’s protocol. RNA purity and quantification were evaluated using the NanoDrop 2000 spectrophotometer (Thermo Scientific, USA). RNA integrity was assessed using the Agilent 2100 Bioanalyzer (Agilent Technologies, Santa Clara, CA, USA). Subsequently, libraries were constructed employing the VAHTS Universal V6 RNA-seq Library Prep Kit, following the manufacturer’s instructions. Transcriptome sequencing and analysis were performed by OE Biotech Co., Ltd. (Shanghai, China).

### RNA-seq and analysis of differentially expressed genes

The libraries were sequenced using the Illumina NovaSeq 6000 platform, generating 150 bp paired-end reads. Initially, raw reads in FASTQ format were processed with Fastp to eliminate low-quality reads, resulting in clean reads. These clean reads were mapped to the mouse genome utilizing HISAT2. FPKM of each gene was calculated and the read counts of each gene were obtained through HTSeq-count. Principal component analysis (PCA) was performed using R (v 4.2.0) to evaluate the biological duplication of samples.

Differential expression analysis was conducted utilizing the DESeq2. *Q* value < 0.05 and fold change > 2 or fold change < 0.5 was set as the threshold for significantly differential expression genes (DEGs). Hierarchical cluster analysis of DEGs was performed using R (v 4.2.0) to demonstrate the expression pattern of genes in different groups and samples. The radar map of the top 30 genes was drawn to show the expression of up-regulated or down-regulated DEGs using R packet ggradar.

Based on the hypergeometric distribution, Kyoto Encyclopedia of Genes and Genomes (KEGG) pathway enrichment analysis of DEGs was performed to screen the significantly enriched term using R (v 4.2.0), respectively. R (v 4.2.0) was used to draw the column diagram, the chord diagram, and the bubble diagram of the significant enrichment term.

### Metabolic flux quantitation

*F_circ_* of a certain metabolite is calculated by the following formula, where *L* is the labeling fraction of this metabolite in the vein, and *R* is the infusion rate of this metabolite in the isotope tracing experiment.


$$\begin{array}{l} F_{circ}=\frac{R\left(1-L\right)}{L} \end{array}$$

The fraction of the mass isotopomer [*M* + *i*] of the nutrient in the sample at steady state is represented by *L*_[*M* + *i*]_ (where 𝑖 ranges from 0 to *N*). Here, *L* represents the proportion of labeled carbon atoms within the nutrient, defined as


$$\begin{array}{l} L=\frac{\overset{N}{\underset{i\;=\;0}{\sum }}i\cdot L_{\left[M+i\right]}}{N} \end{array}$$

With the infusion of a ^13^C-labeled tracer *X*, the normalized labeling of the downstream metabolite *Y* is defined as


$$\begin{array}{l} L{}_{Y}\leftarrow_{X}=\frac{L_{Y}}{L_{X}} \end{array}$$

To ascertain the direct contribution of circulating glucose and glutamine to the tissue lactate and TCA cycle, our calculation was building upon the methodology described by Hui *et al*. [31]. This was achieved by formulating a set of linear equations. As previously defined, the normalized Lactate/TCA labeling in tissue originating from circulating metabolites *X* (*L_Lac/TCA_*_←*X*_) corresponds to the normalized labeling of tissue lactate or citrate in relation to the ^13^C fractional labeling of serum *X*. Considering the metabolic interconversion between glucose and glutamine, the measured Lactate/TCA labeling from *X* can be expressed as the sum of 1*(the direct contribution of nutrient 𝑋 to the Lactate/TCA cycle) and (the direct contribution of nutrient Y to the Lactate/TCA cycle)*[the normalized labeling of Y when X serves as the tracer (*L_Y←X_*)]. The same calculation applies to Lactate/TCA labeling from *Y*. Thus, we were able to derive the linear equations,


<tex−math>

### Relative protein synthesis rate

[U-^13^C] methionine tracing was performed to calculate the relative protein synthesis rate. Mice were fasted from 10:00 a.m. to 6:00 p.m. and refed for 1 h, and then infused 2.5 h, with food in the cage. At the end of infusion, tail blood and tissues were collected. The protein content of the tissue was determined by weighing the tissue powder after liquid nitrogen grinding and the protein extracted from it. Protein was degraded into amino acids by 6 mol/L HCl in 110°C for 8 h, which were measured by LC-MS for ^13^C labeled methionine. The relative protein synthesis rate of the organ was given by the following equation,


$$\begin{array}{lll} & Relative\;protein\;synthesis\;rate\;\; & =\frac{Protein\;content*labeling\;of\;methionine\;in\;tissue\;protein}{Labeling\;of\;methionine\;in\;serum} \end{array}$$

The results were given after normalization to AA diet.

### Clinical sample acquisition and analysis

The clinical samples were collected according to the approval from Ningbo Hangzhou Bay Hospital with No. LY2023-08. In detail, the height and weight of each participant wearing light clothing were measured to the nearest 0.1 cm and 0.1 kg, respectively, using a digital scale and a stadiometer. The BMI was calculated as the body weight (kg) divided by the height (m) squared. All the blood samples were acquired between 07:00 a.m. and 08:00 a.m. after overnight fasting. All participants received 75-g OGTT, and blood samples were collected at 0, 30, 60, 120, and 180 min. The statistical analysis data was adopted at 0-min time point. The blood samples were stored at −20°C on the day of the collection. Blood glucose was measured using the glucose oxidase method. TG was measured by enzymatic assays (Cobas auto analyzer; Roche Diagnostics, Basel, Switzerland). Insulin resistance (IR) was estimated by the homeostasis model assessment of IR (HOMA-IR) method using the following formula: [HOMA-IR = insulin (μU/mL)*glucose (mg/dL)/22.5]. Besides, all patients received the two-step hyperglycemic clamp technique.

For the clinical data, the normality of the data was first assessed using the shapiro.test function, which is based on the Shapiro-Wilk test. The null hypothesis of this test is that the data sample comes from a normal distribution, while the alternative hypothesis is that the data does not follow a normal distribution. Subsequent analyses were performed based on the normality and homoscedasticity of the data. Due to the distribution characteristics of the clinical data in this cohort of type 2 diabetes, several analytes displayed skewness. Thus, non-parametric methods were employed. Correlations were assessed using the Spearman rank correlation coefficient, adjusting for relevant covariates. All tests were two-tailed (^*^P < 0.05; ^**^P < 0.01; ^***^P < 0.001). The data plotting uses the R package called ggplot2, and the data analysis uses the R package ppcor. Clinical data analysis was conducted using the local R environment in R studio (Windows version 4.4.1).

## Supplementary Material

loaf009_suppl_Supplementary_Materials

## Data Availability

All data relevant to the study are included in the article or uploaded as supplementary information. The data used to support the findings of this study will be available on request.
