# Supplementary material for: Dietary sulfur amino acid restriction improves metabolic health by reducing fat mass
Source: Life Metab. 2025 Mar 7;4(3):loaf009. doi: 10.1093/lifemeta/loaf009 (PMC12141819; doi:10.1093/lifemeta/loaf009)
Supplement: loaf009_suppl_Supplementary_Materials [file loaf009_suppl_supplementary_materials.pdf]

Supplementary Table S1. Diet formular of AA, 1/3 BCAA, 1/6 BCAA, 1/3 SAA, 1/6 SAA, and SAAR + Cys, SAAR + Met.

Ingredients	FB-A10021B (AA)	FB-A10022B (1/3 BCAA）	FB-A10022B (1/6 BCAA）	FB-A10022B (1/3 SAA）	FB-A10022B (1/6 SAA）	FB-A10022B (SAAR + Cys）	FB-A10022B (SAAR + Met）
Raw materials	g	g	g	g	g	g	g
L-Arginine	10.00	11.31	11.68	10.50	10.57	10.31	10.24
L-Histidine-HCl-H_2O	6.00	6.79	7.01	6.30	6.34	6.19	6.14
L-Isoleucine	8.00	2.67	1.20	8.40	8.46	8.25	8.19
L-Leucine	12.00	4.00	1.80	12.60	12.68	12.37	12.29
L-Lysine-HCl	14.00	15.84	16.35	14.70	14.80	14.44	14.34
L-Methionine	6.00	6.79	7.01	2.00	0.90	0.90	6.14
L-Phenylalanine	8.00	9.05	9.34	8.40	8.46	8.25	8.19
L-Threonine	8.00	9.05	9.34	8.40	8.46	8.25	8.19
L-Tryptophan	2.00	2.26	2.34	2.10	2.11	2.06	2.05
L-Valine	8.00	2.67	1.20	8.40	8.46	8.25	8.19
L-Alanine	10.00	11.31	11.68	10.50	10.57	10.31	10.24
L-Asparagine-H_2O	5.00	5.66	5.84	5.25	5.28	5.16	5.12
L-Aspartate	10.00	11.31	11.68	10.50	10.57	10.31	10.24
L-Cystine	4.00	4.53	4.67	0.00	0.00	4.12	0.00
L-Glutamic acid	30.00	33.94	35.03	31.50	31.71	30.93	30.72
L-Glutamine	5.00	5.66	5.84	5.25	5.28	5.16	5.12
L-Glycine	10.00	11.31	11.68	10.50	10.57	10.31	10.24
L-Proline	5.00	5.66	5.84	5.25	5.28	5.16	5.12
L-Serine	5.00	5.66	5.84	5.25	5.28	5.16	5.12
L-Tyrosine	4.00	4.53	4.67	4.20	4.23	4.12	4.10
Corn starch	550.50	550.50	550.50	550.50	550.50	550.50	550.50
Maltodextrin	125.00	125.00	125.00	125.00	125.00	125.00	125.00
Cellulose	50.00	50.00	50.00	50.00	50.00	50.00	50.00
Corn Oil	50.00	50.00	50.00	50.00	50.00	50.00	50.00
Mineral mix	35.00	35.00	35.00	35.00	35.00	35.00	35.00
Sodium bicarbonate	7.50	7.50	7.50	7.50	7.50	7.50	7.50
Vitamin mix	10.00	10.00	10.00	10.00	10.00	10.00	10.00
Choline bitrartrate	2.00	2.00	2.00	2.00	2.00	2.00	2.00
Dye	0.05	0.05	0.05	0.05	0.05	0.05	0.05
Total	1000.05	1000.04	1000.05	1000.05	1000.05	1000.05	1000.05

Supplementary Table S2. Diet formular of WD-AA, WD-SAAR, and WD-BCAAR.

Ingredients	TD160186 AA	TD160186-SAAR	TD160186-BCAAR
Raw materials	g	g	g
L-Arginine	10.18	10.52	12.21
L-Histidine-HCl-H_2O	7.84	8.11	9.41
L-Isoleucine	11.51	11.90	1.92
L-Leucine	22.79	23.55	3.80
L-Lysine-HCl	19.65	20.31	23.56
L-Methionine	7.94	1.19	9.52
L-Phenylalanine	13.20	13.64	15.82
L-Threonine	8.56	8.85	10.27
L-Tryptophan	2.36	2.44	2.83
L-Valine	12.99	13.42	2.16
L-Alanine	8.21	8.49	9.85
L-Asparagine-H_2O	12.33	12.74	14.78
L-Aspartate	7.78	8.04	9.33
L-Cystine	1.29	0.00	1.54
L-Glutamic acid	26.57	27.45	31.85
L-Glutamine	22.09	22.83	26.48
L-Glycine	5.43	5.61	6.51
L-Proline	19.80	20.46	23.74
L-Serine	12.03	12.43	14.42
L-Tyrosine	12.73	13.16	15.26
Anhydrous milk fat	210.00	210.00	210.00
sucrouse	340.00	340.00	340.00
corn starch	50.00	50.00	50.00
Maltodextrin	50.00	50.00	50.00
Cellulose	50.00	50.00	50.00
Cholesterol	1.50	1.50	1.50
Mineral Mix, AIN-76 (170915)	35.00	35.00	35.00
Calcium Phosphate $Ca(H2PO4)_2 \cdot H_2O$	8.00	8.00	8.00
Vitamin mix, Teklad (40060)	10.00	10.00	10.00
TBHQ, antioxidant	0.04	0.04	0.04
Total	999.81	999.67	999.81

Supplementary Table S3. Antibodies and qPCR primers.

Antibodies		
Phospho-eIF2alpha (Ser51) (D9G8) XP ® Rabbit mAb	Cell Signaling(CST)	3398T
eIF2alpha (D7D3) XP ® Rabbit mAb	Cell Signaling(CST)	5324T
Phospho-p70 S6 Kinase (Thr389) (108D2) Rabbit mAb	Cell Signaling(CST)	9234T
p70 S6 Kinase (E8K6T) XP® Rabbit mAb	Cell Signaling(CST)	34475T
PERK (C33E10) Rabbit mAb	Cell Signaling(CST)	3192T
Phospho-PERK (Thr980) (16F8) Rabbit mAb	Cell Signaling(CST)	3179T
AMPKalpha (D5A2) Rabbit mAb	Cell Signaling(CST)	5831T
Phospho-AMPKalpha (Thr172) (40H9) Rabbit mAb	Cell Signaling(CST)	2535T
qPCR		
mRNA	Forward Sequence 5′-3′	Reverse Sequence 5′-3′
PPAR-γ	GTACTGTCGGTTTCAGAAGTGCC	ATCTCCGCCAACAGCTTCTCCT
CIDEA	GGTGGACACAGAGGAGTTCTTTC	CGAAGGTGACTCTGGCTATTCC
DIO2	GGTGGTCAACTTTGGTTCAGCC	AAGTCAGCCACCGAGGAGAAC
UCP1	GCTTTGCCTCACTCAGGATTGG	CCAATGAACACTGCCACACCTC

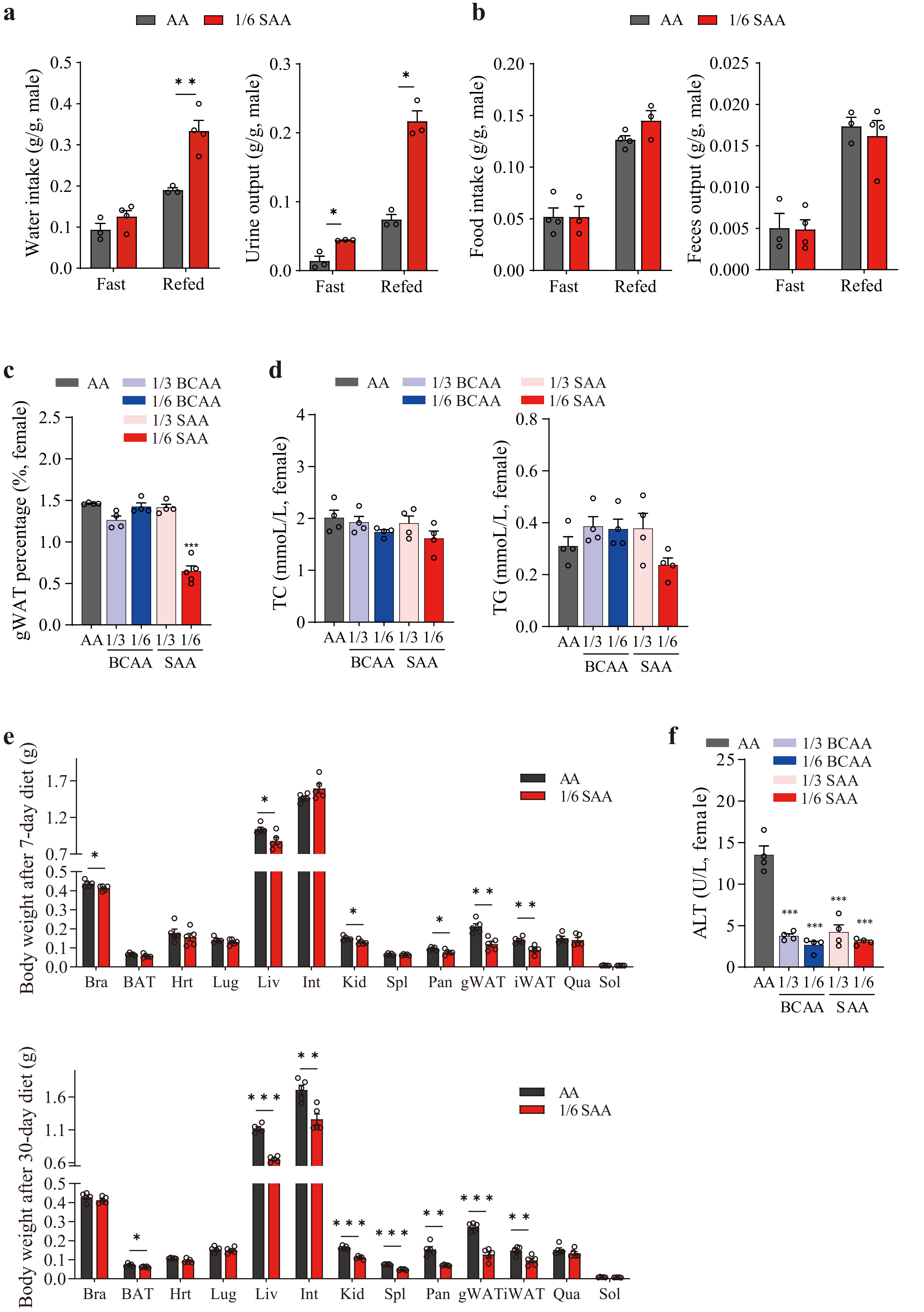

Supplementary Figure S1

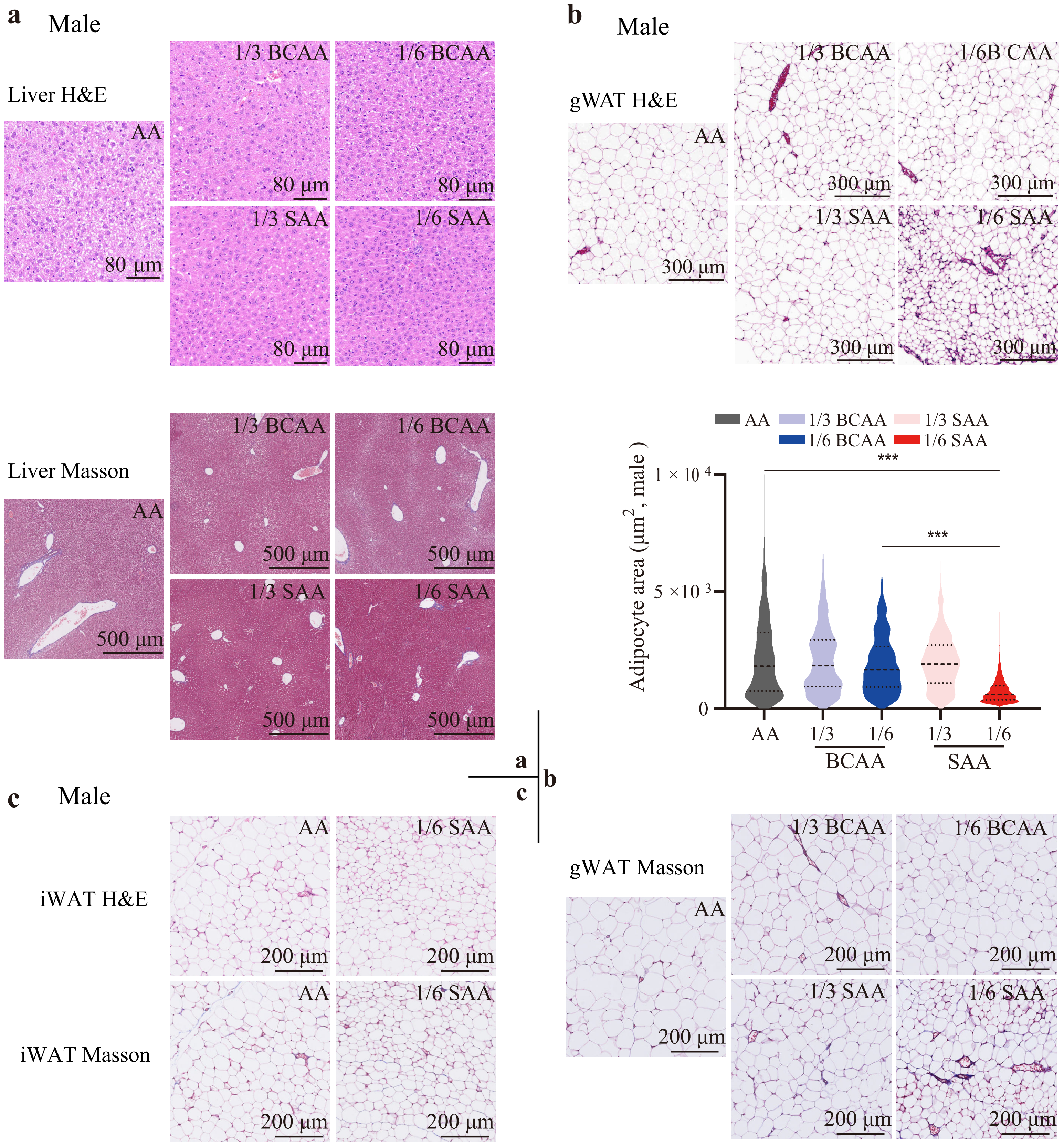

Supplementary Figure S2

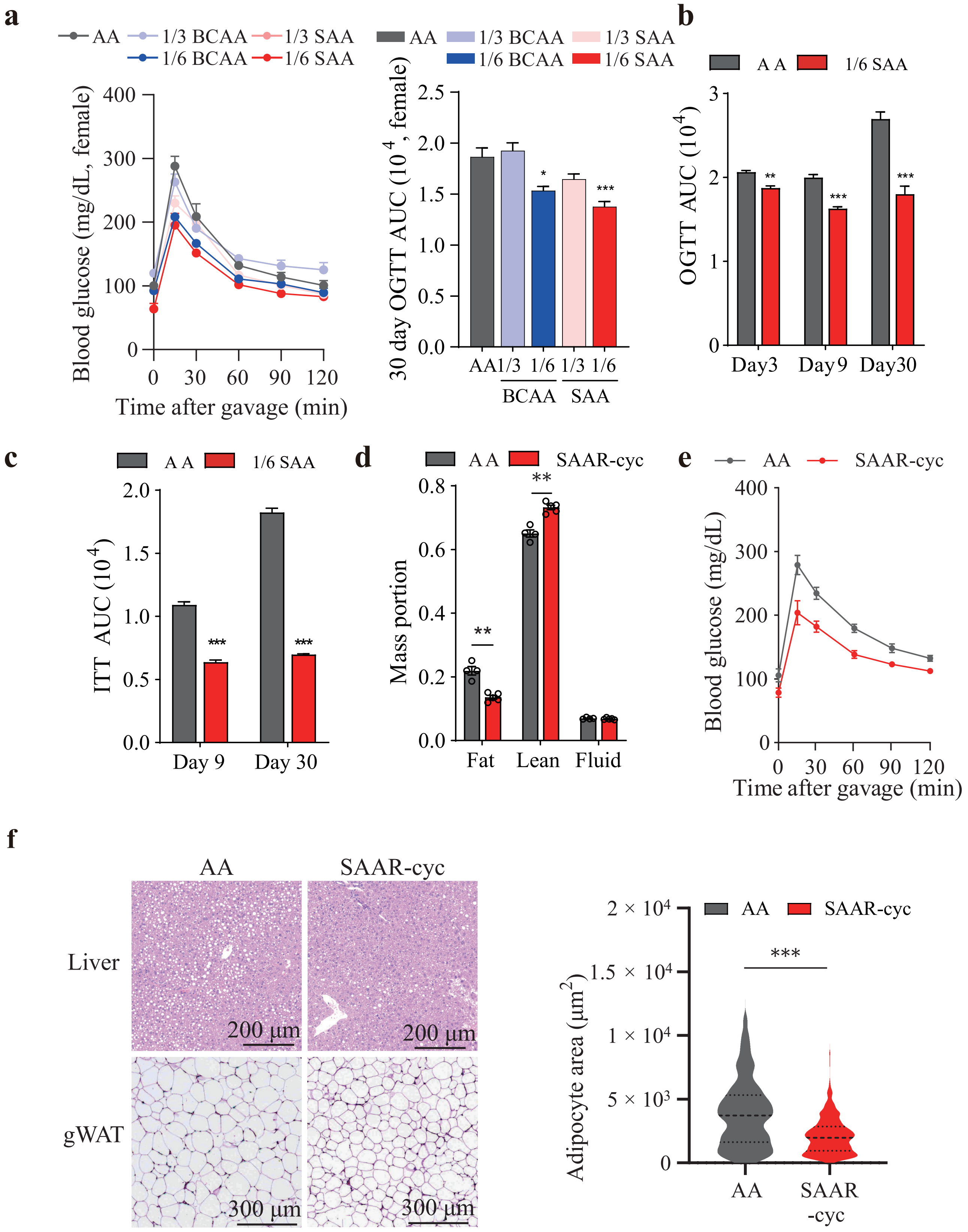

Supplementary Figure S3

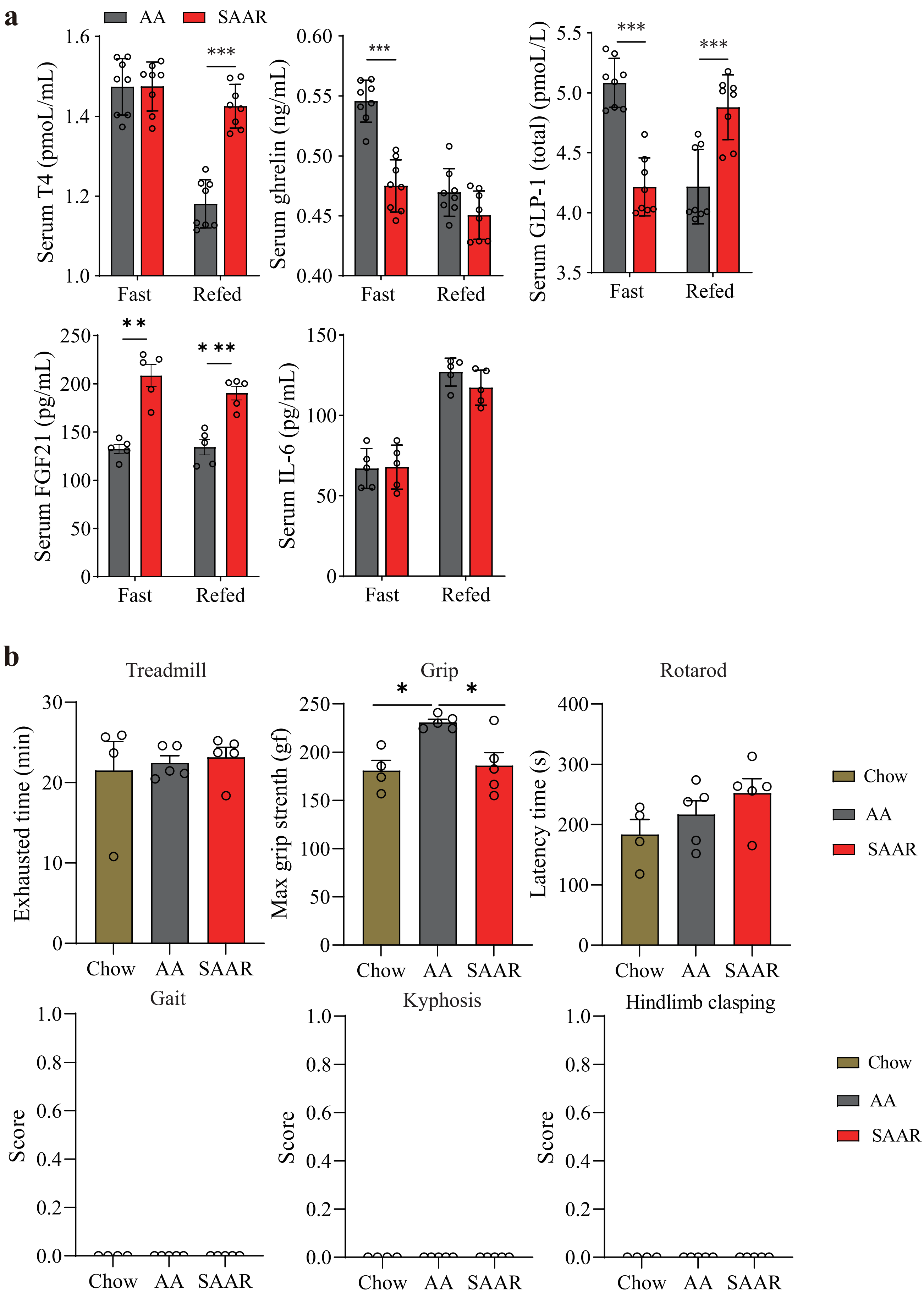

Supplementary Figure S4

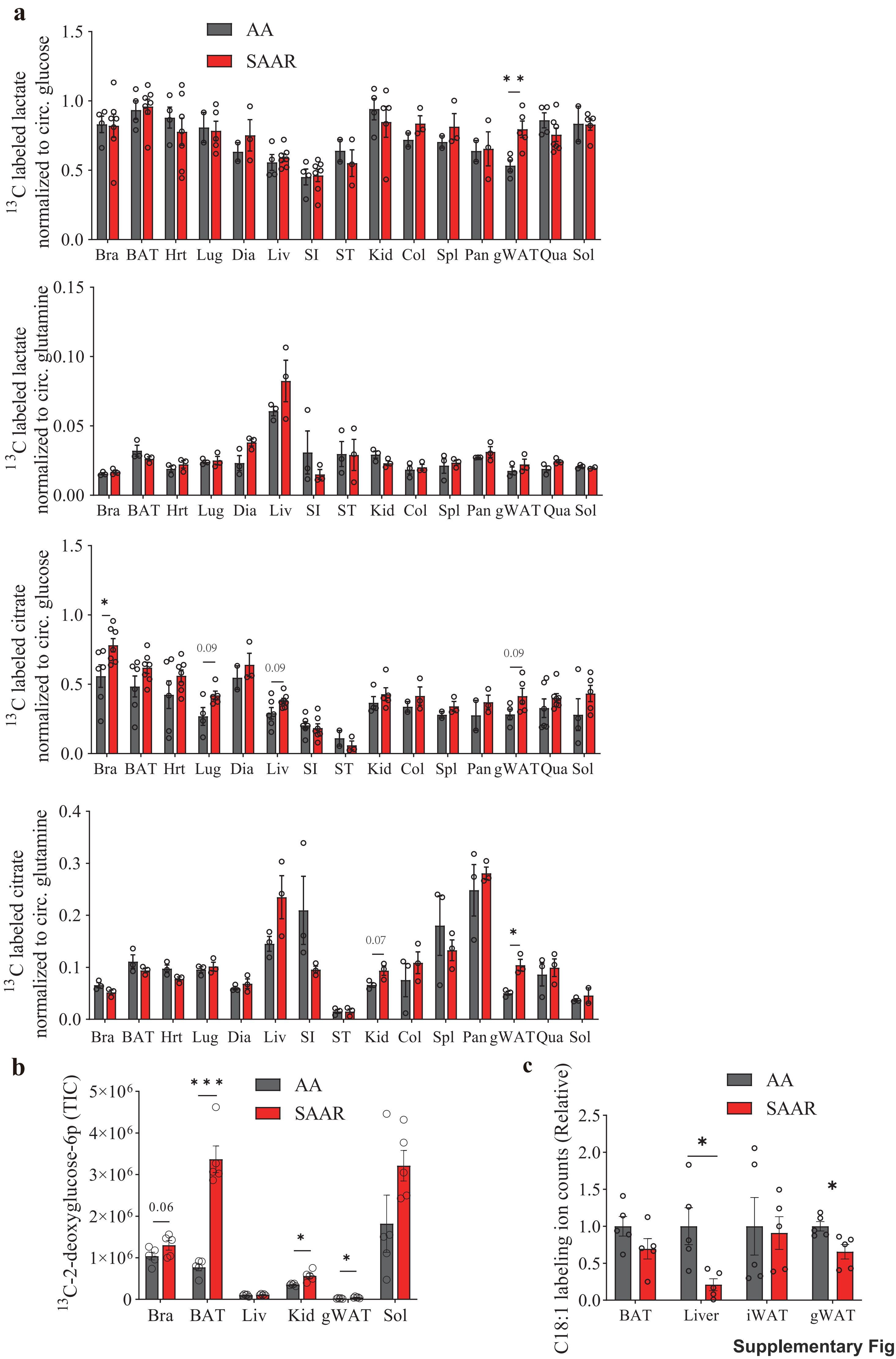

Supplementary Figure S5

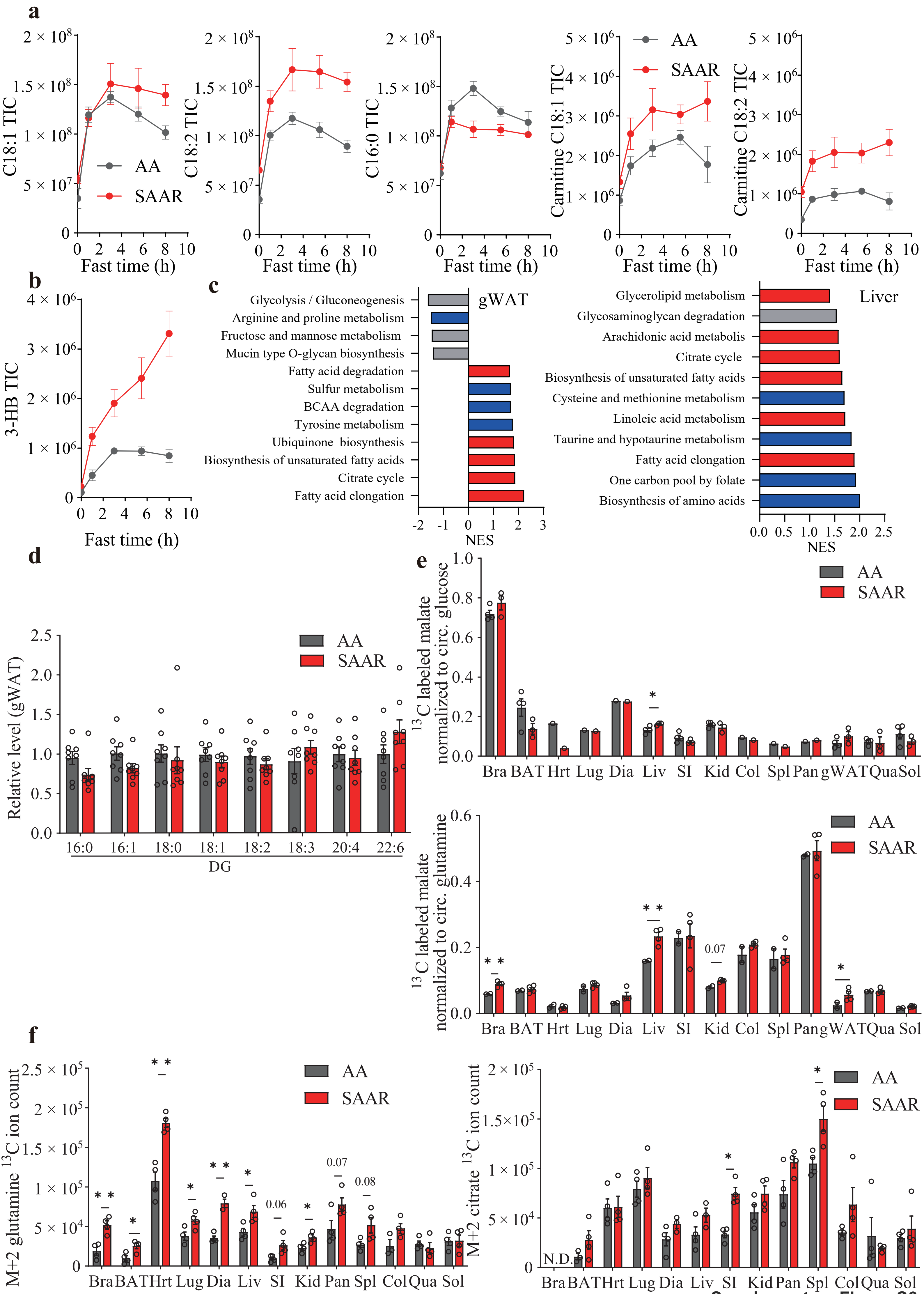

Supplementary Figure S6

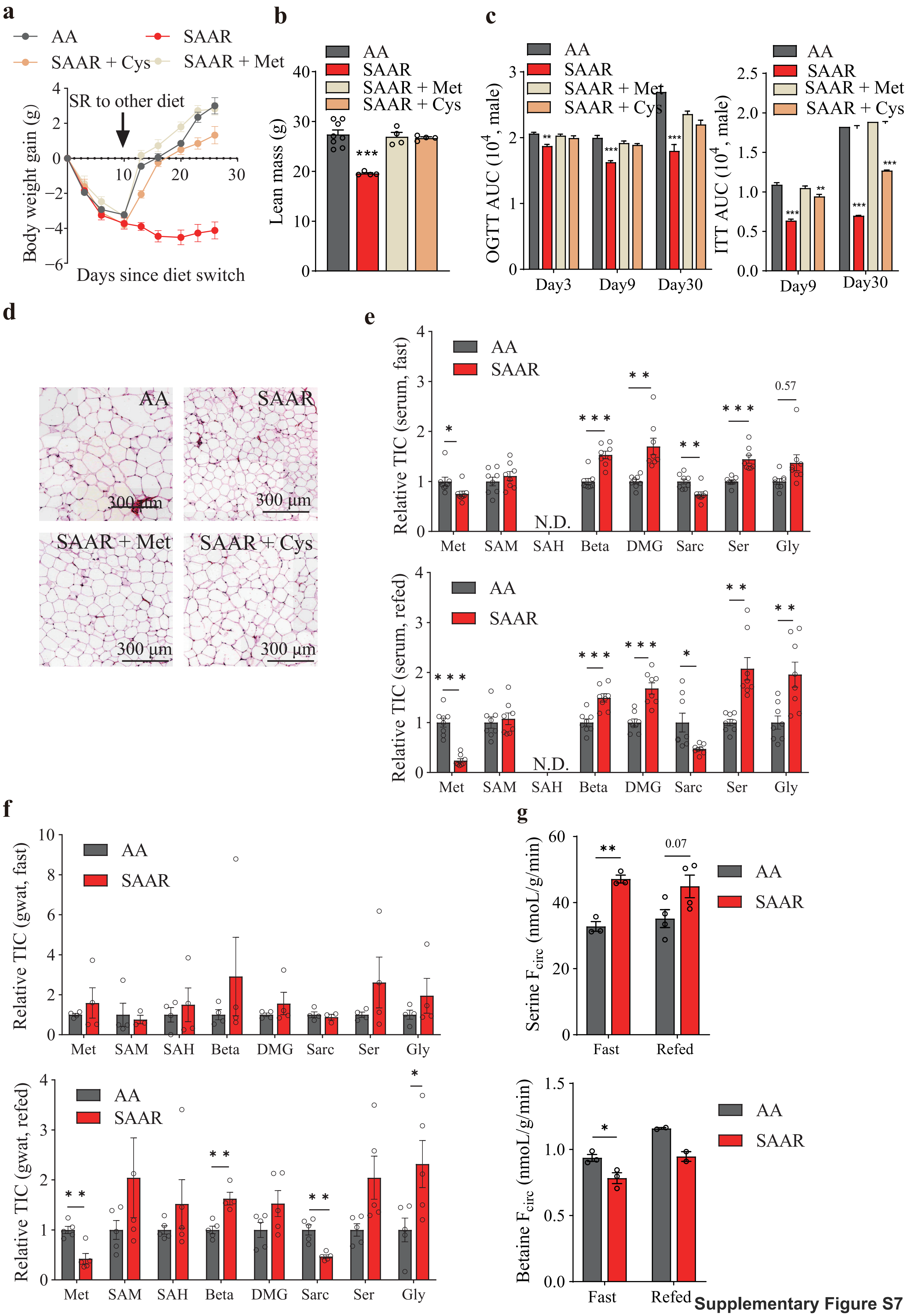

Supplementary Figure S7

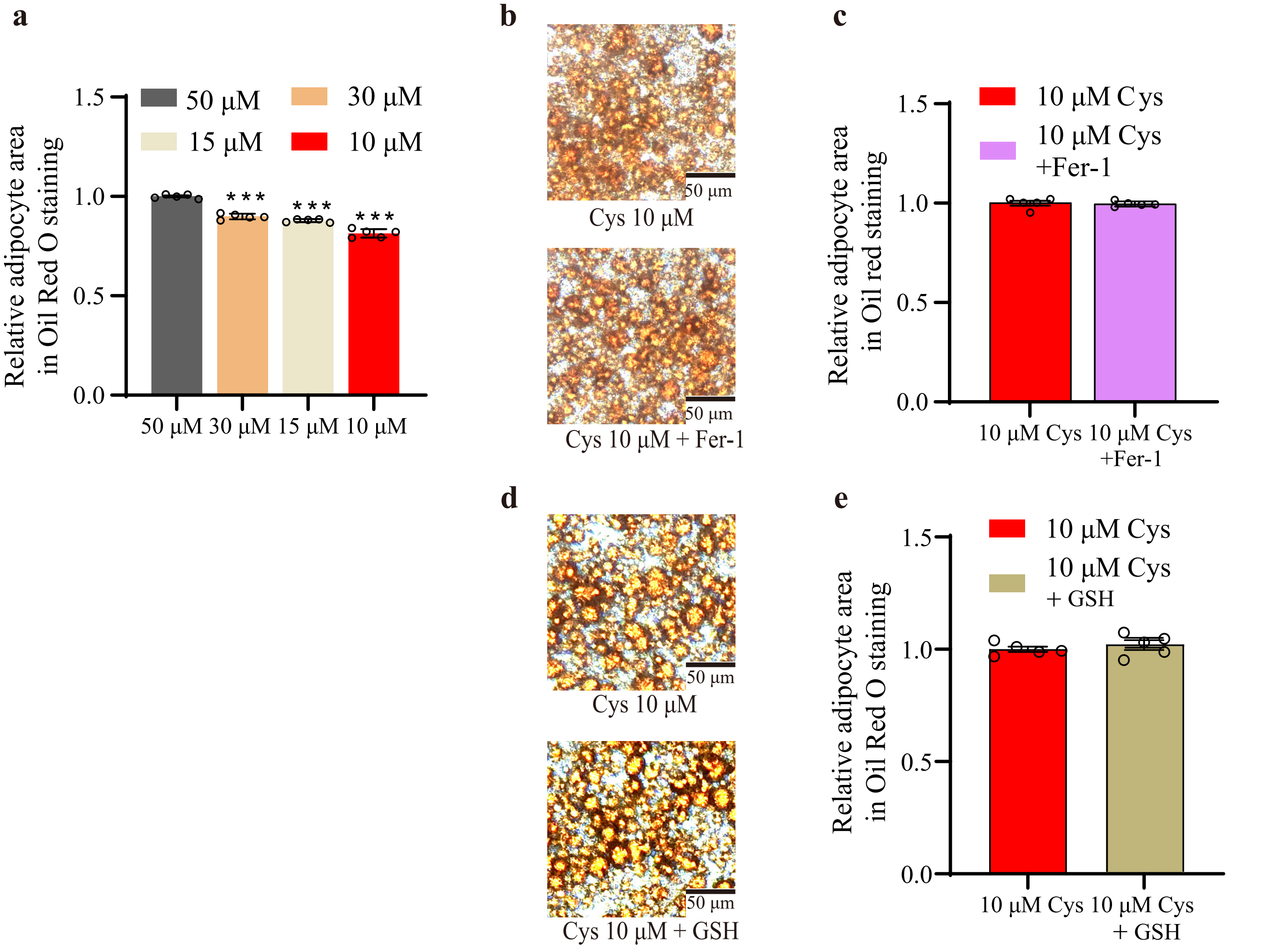

Supplementary Figure S8

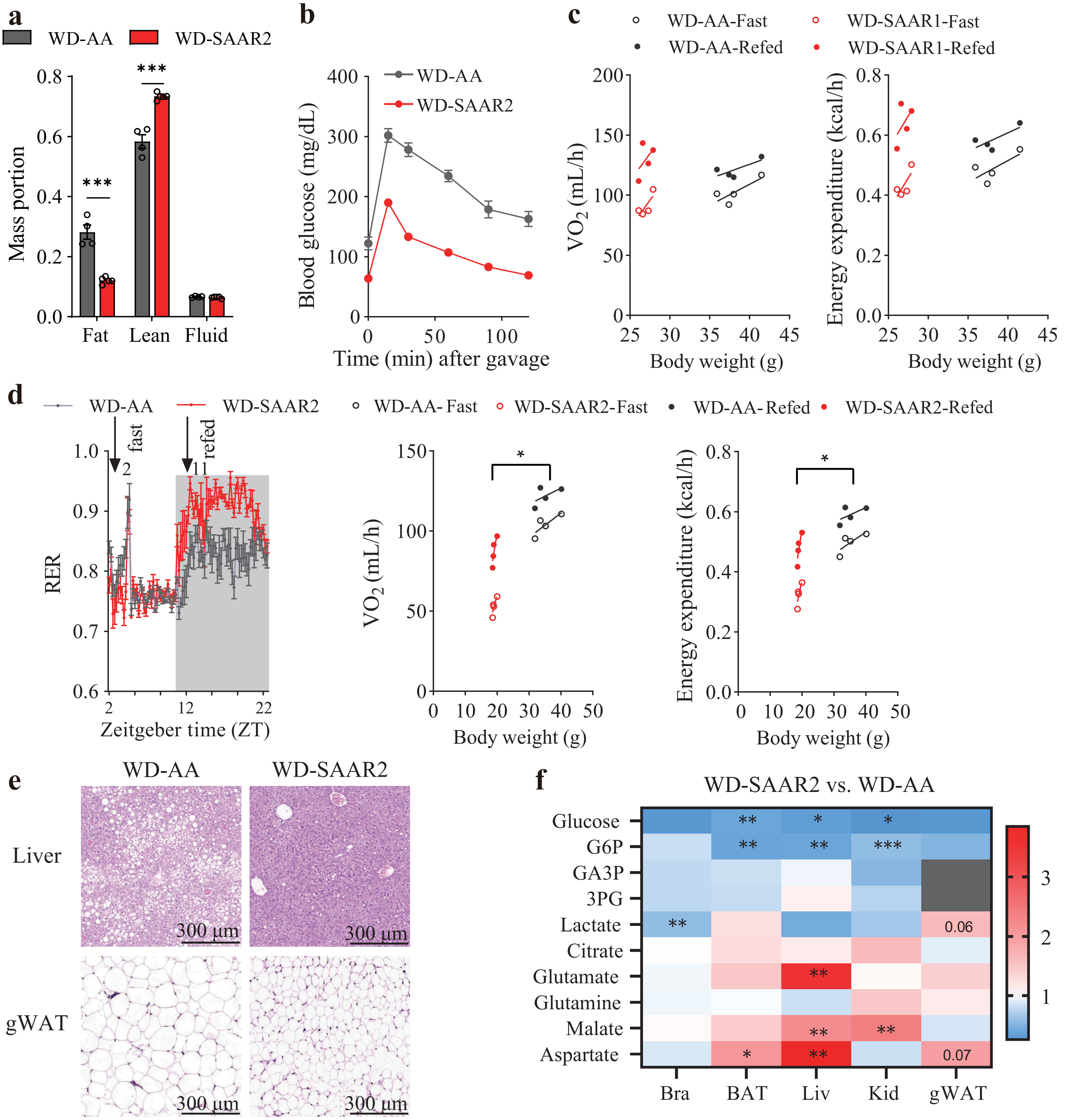

g

Clinical information	Cohort<br>Type 2 diabetes mellitus<br>(*n* = 41)
Age, median (IQR)	56 (48−65)
BMI, median (IQR)	23.7 (22.1−26.0)
Insulin, median (IQR)	10.8 (9.0−19.1)
TG, mmol/L, median (IQR)	1.21 (0.9−1.64)
Glucose, mmol/L, median (IQR)	6.81(5.86−7.33)
HOMAir	3.46 (2.39−5.06)
Cystine, Intensity, median (IQR)	1264093 (1066261−1450918)
For continuous variables, values are presented as median (quartile)	

Supplementary Figure S9

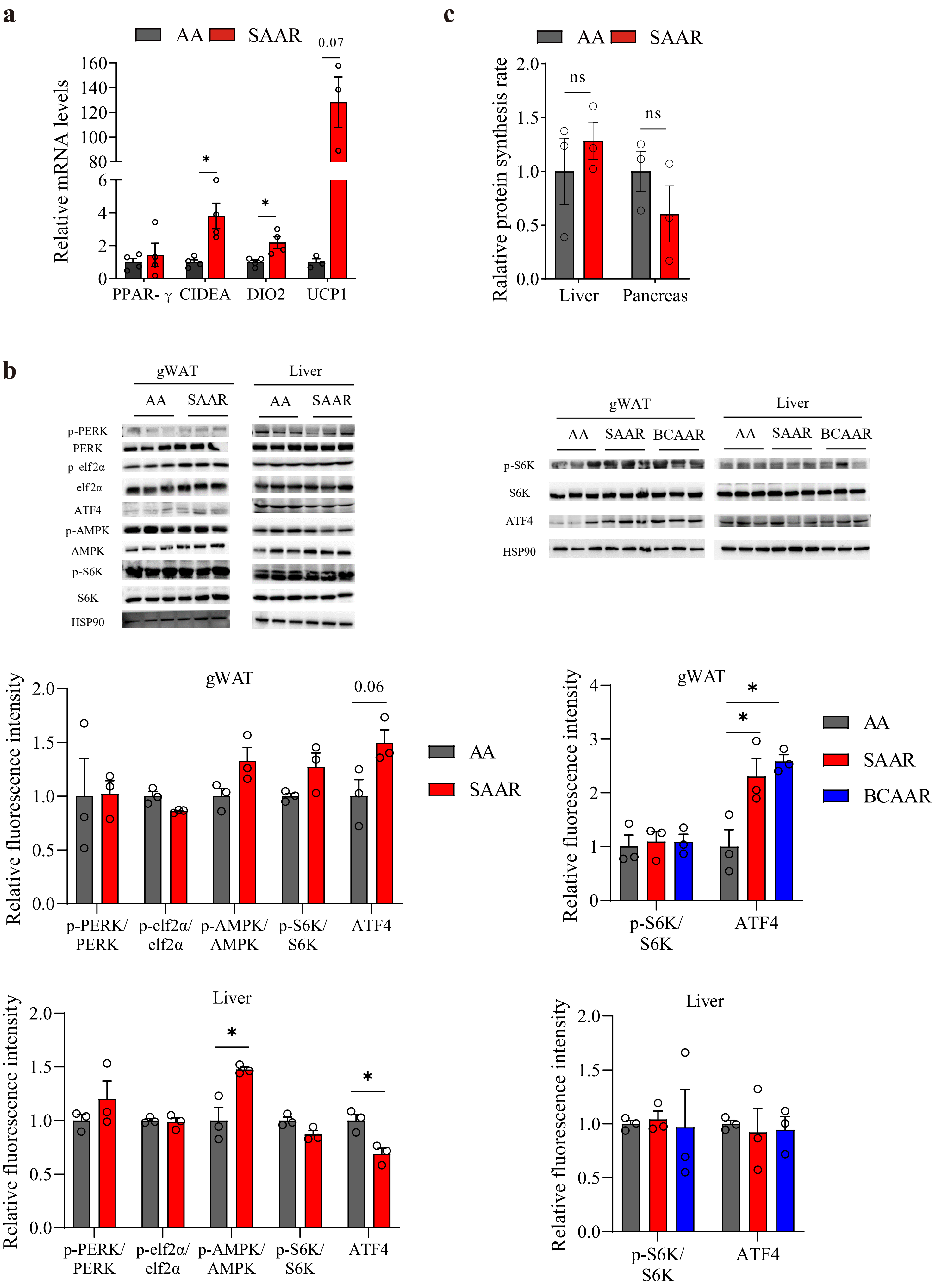

Supplementary Figure S10

Supplementary Figure S1 (a and b) Water intake, urine output, food intake and feces output of mice in *ad lib* fasting and feeding periods, normalized to body weight. Water intake: AA, *n* = 3; 1/6 SAA, *n* = 4; urine output: AA, *n* = 3; 1/6 SAA, *n* = 3; food intake: AA, *n* = 4; 1/6 SAA, *n* = 3; feces output: AA, *n* = 3, 1/6 SAA, *n* = 4. (c) Weight percentage of gonadal fat in female mice fed with different diets. Female: AA, *n* = 4; 1/3 BCAA, *n* = 4; 1/3 SAA, *n* = 4; 1/6 BCAA, *n* = 4; 1/6 SAA, *n* = 5. (d) Circulatory levels of total triglycerides (TG) and cholesterol (TC) in female mice fed with different diets. *n* = 4. (e) Organ weight of mice after 7 days and 30 days of feeding with AA and 1/6 SAA diets. *n* = 5. (f) Circulatory levels of alanine aminotransferase (ALT) in female mice fed with different diets. *n* = 4. Data are expressed as mean ± SEM and statistical differences were calculated by unpaired two-tail *t*-test if unspecified. *P < 0.05; **P < 0.01; ***P < 0.001.

Supplementary Figure S2 (a) H&E staining of liver from male mice feeding with different diets after 30 days. (b) H&E staining, Mmasson staining, and adipocyte area statistics of gWAT from male mice fed with different diets after 30 days. (c) H&E staining and Masson staining of iWAT from mice fed with AA and 1/6 SAA diets after 30 days. Data are expressed as mean ± SEM and statistical differences were calculated by unpaired two-tail *t*-test and one-way ANOVA with Dunnett's correction. *P < 0.05; **P < 0.01; ***P < 0.001.

Supplementary Figure S3 (a) Circulatory glucose levels and its corresponding area under the curve in female mice gavaged with 1g/kg glucose after 16 h of fasting. Female: AA, *n* = 4; 1/3 BCAA, *n* = 4; 1/3 SAA, *n* = 4; 1/6 BCAA, *n* = 5; 1/6 SAA, *n* = 5. Data statistical differences were calculated by one-way ANOVA with Dunnett’s correction. (b) Circulatory glucose level corresponding area (at day 3, day 9, and day 30) under the curve in male mice gavaged with 1g/kg glucose after 16 h of fasting. *n* = 4. (c) Circulatory glucose level corresponding area (at day 9 and day 30) under the curve in mice injected with 1U/kg insulin after 4 h of fasting. *n* = 3. (d) Percentage of mass portions for mice subjected to dietary cycling and those continuously feeding the AA diet at the end point. AA, *n* = 4; SAAR-cyc, *n* = 5. (e) Circulatory glucose levels in mice subjected to dietary cycling and those continuously feeding the AA diet at the 3rd day point after switching to AA diet, gavaged with 1g/kg glucose after 16 h of fasting. AA, *n* = 4; SAAR-cyc, n = 5. (f) H&E staining of livers and gWAT, and adipocyte area statistics of gWAT from mice fed with AA or SAAR-cycle. Data are expressed as mean ± SEM and statistical differences were calculated by unpaired two-tail *t*-test if unspecified. *P < 0.05; **P < 0.01; ***P < 0.001.

Supplementary Figure S4 (a) Circulatory T4, ghrelin, GLP-1, FGF21, and IL-6 levels in fasting and refed periods. T4, ghrelin, GLP-1: n = 8; FGF21, IL-6: n = 5. (b) Treadmill test, grip test, and rotarod test were performed to measure exercise capability of mice; qualitative assessment of gait, kyphosis and hindlimb clasping. Normal, score = 0; abnormal, score = 1. Chow, n = 4; AA, n = 5; SAAR, n = 5.

Supplementary Figure S5 (a) ^{13}C enrichment of tissue lactate/citrate from [U-^{13}C] glucose and [U-^{13}C,^{15}N] glutamine tracing. n as indicated for each tracer in different organs. (b) Total ion counts (TIC) of [U-^{13}C] 2-deoxyglucose phosphate in different tissues. n = 5. (c) Relative labeling ion counts of C18:1 in different tissues. n = 5. Data are expressed as mean ± SEM and statistical differences were calculated by unpaired two-tail t-test if unspecified. *$P < 0.05$; **$P < 0.01$; ***$P < 0.001$.

Supplementary Figure S6 (a and b) Total ion counts of C18:1, C18:2, C16:0, carnitine C18:1, carnitine C18:2 (a) and 3-HB (b) in serum from mice fed with AA diet and SAAR diet in time-course fasting period. n = 5. (c) RNA-seq pathway analysis of gWAT and liver from mice fed with AA diet and SAAR diet after 8 h of fasting. (d) Relative abundance of FA in DG species from lipidomic analysis of gWAT data after 8 h of fasting. n = 8. (e) ^{13}C enrichment of malate in different tissues from mice fed with different diets, normalized to circulatory ^{13}C labeled glucose or ^{13}C labeled glutamine. Tissue was collected after 2.5 h of [U-^{13}C] glucose and [U-^{13}C,^{15}N] glutamine tracing. n as indicated for each tracer in different organs. (f) ^{13}C ion counts of M+2 citrate and M+2 glutamine in multiple tissues for mice fed with AA diet and SAAR diet. Tissues were collected after 30 min retro-orbital injection of [U-^{13}C] palmitic acid. n = 4. Data are expressed as mean ± SEM and statistical differences were calculated by unpaired two-tail t-test if unspecified. *$P < 0.05$; **$P < 0.01$; ***$P < 0.001$.

Supplementary Figure S7 (a) Weight gain for mice fed with SAAR diet for 10 days, switching to indicated diets for 15 days. n = 4. (b) Lean mass of mice fed with different diets. n = 4. (c) Area under the curve of circulatory glucose levels (at day 3, day 9, and day 30) gavaged with 1g/kg glucose after 16 h of fasting. n = 4. (c) Area under the curve of circulatory glucose levels (at day 9 and day 30) injected with 1 U/kg insulin after 4 h of fasting. n = 4. (d) H&E staining of gWAT from mice fed with different diets. (e) Relative total ion counts (TIC) of metabolites in serum from mice fedg with AA diet and SAAR diet, normalized to AA diet. n = 12. (f) TIC of metabolites in gWAT from mice fed with AA diet and SAAR diet, normalized to AA diet. Fast, n = 4; refed, n = 5. (g) The turnover rate of serine and betaine in mice. All fast, n = 3; refed_serine_AA, n = 3; refed_serine_SAAR, n = 4; all refed_betaine, n = 2. Data are expressed as mean ± SEM and statistical differences were calculated by unpaired two-tail t-test if unspecified and one-way ANOVA with Dunnett's correction. *$P < 0.05$, **$P < 0.01$, ***$P < 0.001$.

Supplementary Figure S8 (a) Relative adipocyte area in Oil Red O staining from SVF cells cultured with different concentrations of cystine (50 μmol/L, 30 μmol/L, 15 μmol/L, and 10 μmol/L). *n* = 5. (b) Oil Red O staining of SVF cells cultured in medium with and without ferroptosis inhibitor at 10 μmol/L cystine for 3 days. (c) Relative adipocyte area in Oil Red O staining from SVF cells cultured with and without ferroptosis inhibitor at 10 μmol/L cystine for 3 days. *n* = 5. (d) Oil Red O staining of SVF cells cultured in medium with and without GSH at 10 μmol/L cystine for 3 days. (e) Relative adipocyte area in Oil Red O staining from SVF cells cultured with and without GSH at 10 μmol/L cystine for 3 days. n = 5. Data are expressed as mean ± SEM and statistical differences were calculated by unpaired two-tail *t*-test if unspecified. ***$P < 0.001$.

Supplementary Figure S9 (a) Percentage of mass portions of mice fed with WD-AA diet and WD-SAAR2 diet. WD-AA, *n* = 4; WD-SAAR2, *n* = 5. (b) Circulatory glucose levels in mice fed with WD-AA diet and WD-SAAR2 diet gavaged with 1 g/kg glucose after 16 h of fasting. WD-AA, *n* = 4; WD-SAAR2, *n* = 5). (c) Oxygen inhale and energy expenditure rates of mice fed with WD-AA diet and WD-SAAR1 diet in fasting and refed periods, normalized via ANCOVA. *n* = 5. (d) Respiratory exchange ratio (RER), oxygen inhale rates, and energy expenditure rate of mice fed with WD-AA diet and WD-SAAR2 diet. *n* = 4−5. (e) H&E staining of livers and gWAT from mice fed with WD-AA diet and WD-SAAR2 diet. (f) Fold change (WD-SAAR2/WD-AA) of ^{13}C TCA cycle metabolites in mice gavaged with 1g/kg U-^{13}C glucose. (g) Clinical database information. For continuous variables, values are presented as median (quartile). Data are expressed as mean ± SEM and statistical differences were calculated by unpaired two-tail *t*-test if unspecified. *$P < 0.05$; **$P < 0.01$; ***$P < 0.001$.

Supplementary Figure S10 (a) Relative mRNA levels of PPAR-γ, CIDEA, DIO2 and UCP1 in iWAT from mice after 30 days of feeding with AA and SAAR diets. PPAR-γ, CIDEA, DIO2: *n* = 4; UCP1: *n* = 3. (b) Western blot and relative fluorescence intensity in western blot of gWAT and liver from mice after 30 days (left) and 60 days (right) of feeding with different diets, normalized to AA diet. Data of phosphorylated protein were divided by the original protein. *n* = 3. (c) Relative protein synthesis rate in liver and pancreas of mice infused by [U-^{13}C] methionine.
